# A Versatile Open-Source Printhead for Low-Cost 3D Microextrusion-Based Bioprinting

**DOI:** 10.3390/polym12102346

**Published:** 2020-10-13

**Authors:** Andres Sanz-Garcia, Enrique Sodupe-Ortega, Alpha Pernía-Espinoza, Tatsuya Shimizu, Carmen Escobedo-Lucea

**Affiliations:** 1 Division of Pharmaceutical Biosciences, University of Helsinki, Viikinkaari 5 E (P.O. Box 56), 00014 Helsinki, Finland; andres.sanz-garcia@helsinki.fi (A.S.-G.); enrique.sodupeo@unirioja.es (E.S.-O.); 2 Institute of Advanced Biomedical Engineering and Science, Tokyo Women’s Medical University, 8-1 Kawada-cho, Shinjuku-ku, Tokyo 162-8666, Japan; shimizu.tatsuya@twmu.ac.jp; 3 Department of Mechanical Engineering, University of La Rioja, San José de Calasanz 31, Edificio Departamental, 26004 Logroño, Spain; alpha.pernia@unirioja.es

**Keywords:** bioprinting, microextrusion, tissue engineering, bioink, open-source, stem cells

## Abstract

Three-dimensional (3D) bioprinting promises to be essential in tissue engineering for solving the rising demand for organs and tissues. Some bioprinters are commercially available, but their impact on the field of Tissue engineering (TE) is still limited due to their cost or difficulty to tune. Herein, we present a low-cost easy-to-build printhead for microextrusion-based bioprinting (MEBB) that can be installed in many desktop 3D printers to transform them into 3D bioprinters. We can extrude bioinks with precise control of print temperature between 2–60 °C. We validated the versatility of the printhead, by assembling it in three low-cost open-source desktop 3D printers. Multiple units of the printhead can also be easily put together in a single printer carriage for building a multi-material 3D bioprinter. Print resolution was evaluated by creating representative calibration models at different temperatures using natural hydrogels such as gelatin and alginate, and synthetic ones like poloxamer. Using one of the three modified low-cost 3D printers, we successfully printed cell-laden lattice constructs with cell viabilities higher than 90% after 24-h post printing. Controlling temperature and pressure according to the rheological properties of the bioinks was essential in achieving optimal printability and great cell viability. The cost per unit of our device, which can be used with syringes of different volume, is less expensive than any other commercially available product. These data demonstrate an affordable open-source printhead with the potential to become a reliable alternative to commercial bioprinters for any laboratory.

## 1. Introduction

Organ transplantation remains as the unique viable option for some of the most severe organ malfunctions [1], but the shortage of donors makes this therapy utterly unsustainable on a global scale [2]. The gap between the number of donors and patients in the waiting lists has steady grown steadily since the 90 s [3]. Tissue engineering (TE) is a new multidisciplinary field intended to solve the current lack of organs. Even if TE has shown significant progress in the creation of some avascular tissues such as bone or cartilage, engineering functional organs have been left mostly unrealized [4]. Among other biofabrication techniques, 3D bioprinting represents an exciting new research direction. This is a layer-by-layer technology capable of depositing cells, biomaterials, and biological molecules in complex 3D constructs [5,6]. Microextrusion-based bioprinting (MEBB) is the most common technique due to its great deposition rate, affordability, flexibility, and number of compatible bioinks [7]. During the last decade, efforts have been focused on expanding MEBB capabilities to produce in vitro tissue constructs of a clinically relevant size for cardiac [8], dermal [9], cartilage [10], and bone [11] regeneration, among many others [5]. Despite these achievements, critical challenges for MEBB [12] are still high shear stress on cells and low print resolution that always generates features greater than 100 µm (average resolution around 200 µm) [13]. On the contrary, other techniques such as inkjet-based bioprinters can reach print resolutions closer to 50 µm [14]. Lastly, their high cost is another important limitation for this technology in many laboratories. 

Except few custom-built 3D printers [7,12,15,16], commercial MEBB systems are mostly standardized products nowadays [17,18,19]. Some commercial models such as the 3D-Bioplotter^®^ from Envisiontec [20], or 3DDiscovery^®^ and BioFactory^®^ from RegenHU [21,22] are considered the best high-end standardized equipment with positional precision up to 1 µm at prohibitive cost. Recently, new companies such as Allevi (formerly BioBots), Rokit, and Cellink^®^ have brought out more cost-efficient solutions (US$10,000–40,000) positional precision between 10 and 50 µm [23]. In view of recent publication using these more affordable 3D bioprinters, acceptable print resolutions were achieved even considering the significant leap in terms of positional precision between them and high-end proprietary MEBB systems [24,25,26]. This opens the door to search open-source alternative approaches that avoid the proprietary nature of commercial bioprinters and provide similar service at a much lower cost. 

The initial open-source bioprinters such as Fab@Home [27] used mechanically driven pistons. In the literature, there are some examples of desktop 3D fused deposition modeling (FDM) printers that were modified by installing custom-built gear drive extruders for 3D bioprinting [28,29]. Their positional precision is approximately 50 μm (Table S1), far from the high-end 3D printers but at a reduced cost (US$2000). But pneumatic actuators are now preferred as they provide enough precision and sterility at very low cost [30]. However, it remains unclear whether all these open-source alternatives such as the do-it-yourself (DIY) Reprap [31] will be able to fulfill the strict requirements of 3D bioprinting.

Three main biopolymers for 3D bioprinting used in this article are the main components of current bioprinting works: poloxamer, gelatin, and alginate [32]. Like other published works, the poloxamer is very useful for calibration and initial tests, however, it can be used in different bioprinting strategies as sacrificial material [33]. Perhaps, porcine gelatin is the most widely used biopolymer for bioprinting, particularly the one functionalized with methacrylate groups (GelMA). Mixing GelMA and a photoinitiator can make the bioink photo cross-linkable and very useful for various applications [34]. The popular alginate has the main drawback of lack of intrinsic cell-adhesive motifs [35]. The combination of gelatin and alginate in different concentrations [36] is also a standard solution to create in vitro 3D printed models, such as scaffolds to maintain rat Schwann cells activity [37] and constructs of concentrated alginate/gelatin with nano-apatite coating [38], among many others.

We believe that flexible open-source MEBB systems while low in cost are still precise in controlling the print parameters area still needed. These systems might be based on open-source 3D FDM printing projects. To this aim, we present a modular printhead compatible with the X-carriage of many of these FDM printers and capable of controlling the temperature of syringes with different volumes. Herein, we prove the versatility of our system by its installation into three open-source 3D printers without tedious modifications. Finally, to assess the performance, print resolution, and cell viability of our printhead, different calibration models were printed using well-known bioinks following a new benchmark methodology to assess the performance, print resolution, and cell viability.

## 2. Materials and Methods 

### 2.1. Printhead Design and Fabrication

The printhead design follows the particular geometry of three volumes of syringe barrels (Nordson EFD Optimum, USA): 3, 5, and 10 mL. A general exploded view using a 3D modeling software (SolidWorks, Dassault Systèmes, France) with all the printhead components is shown in Figure S1. Briefly, the syringe is surrounded by an aluminum (Al) block (6061, Almacenes Generales R. Andrade S.L., Pontevedra, Spain) customized (Figure S2). Two Peltier modules (TES1-12704; Hebei Co. Ltd., Shanghai, China) in contact with the Al block control the continuous heating/cooling operations of the printhead. The Peltiers are cooled by Al heat sinks (FANP1003LD; StarTech.com Ltd., Northampton, UK), and all together are braced to the carcass of the printhead using 3D printed clamps. An Al plate is placed between the heat sinks and the Peltiers to facilitate their adjustment. Two EPCOS 100K thermistors (TDK Electronics AG -previously EPCOS, Munich, Germany) are inserted into the Al block and the heat sinks to measure the printhead temperature. The printing pressure is controlled pneumatically using a 12 Vdc solenoid valve (SMC VT307-6DZ1-01F-Q, SMC Company, Tokyo, Japan) and a pressure regulator (SMC ARP20K-N01BG-1Z, SMC Company, Tokyo, Japan). The syringes are fixed to the printhead using a printed cover and screwed (M3) to the upper part of the printhead carcass. Table S2 summarizes the complete list of materials, their costs, and providers.

Some parts were saved as stereolithography (STL) files. All of them are publicly available at the NIH 3D Print Exchange repository (https://3dprint.nih.gov/users/telab). These parts were processed with Slic3r software to produce G-code data, and printed in acrylonitrile butadiene styrene (ABS) (ABSTech, FFFWorld, Spain). All the printhead components were then assembled together as previously described (Figure S3). Different carriages were finally created to install the printhead in different open-source desktop 3D printers, including one for the Witbox2 with capacity for four printheads to develop a multi-material bioprinter. 

### 2.2. Specifications and Modifications of the 3D Printers

The experiments were performed using three desktop open-source 3D printers: Witbox2 (BQ, Navarra, Spain), RepRap BCN3D+ (BCN3D Technologies, Barcelona, Spain) and Sigma (BCN3D Technologies, Barcelona, Spain). RepRap BCND3D+ 3D printer was purchased as a kit, while Witbox2 and Sigma were already-assembled units. Manufacturer’s specifications established positional precisions up to 20, 12.5, and 50 μm for Witbox2, Sigma and BCN3D+, respectively (Table S1). All 3D printers use a Cartesian dimensional coordinate system, with some variations in their XYZ-axes movements. Witbox2 and Sigma 3D printheads can move along the XY horizontal plane whereas the printing platform moves vertically (Z-axis). On the contrary, BCN3D+ printhead moves on the XZ plane while the printing platform moves along the Y-axis.

The open-source electronics for the printers included an Arduino microcontroller based on ATmega2560 (Mega 2560 rev3, Arduino.cc, Italy) and a RepRap Arduino Mega Pololu Shield v1.4 (RAMPS 1.4, Ultimachine, South Pittsburg, TN, USA) connected to a 12 Vdc/30 A power supply. However, open-source Rumba (Reprap Universal Mega Board with Allegro driver, RepRapDiscount, Hong Kong, China) or RAMBo (RepRap Arduino Mega-compatible mother Board, Ultimachine, South Pittsburg, TN, USA) are fully compatible alternatives. Figure S4 depicts a representative scheme of the electronics used. Briefly, Peltier units and the solenoid valve were connected to the MOSFET (metal-oxide-semiconductor field-effect transistor) terminals in the boards. Standard NEMA 17 stepper motors were installed in all the printers. 

A publicly available modification of the open-source Marlin firmware (v1.1, http://marlinfw.org) was loaded into the printer electronics to control the cooling/heating process and open/close the valves [39]. The Repetier-Host free software (V0.56, Willich, Germany), which incorporates all the printing parameters, was used to control the printing process as well as the load of the G-code of the models [40].

### 2.3. Thermal Performance of the Printhead

Operating printhead temperatures, stability, and heating/cooling times for three different sizes of syringes were evaluated. Starting from 22 °C, various heating and cooling cycles were performed to reach the target temperatures of 37 °C and 5 °C, respectively. Two thermistors located at the Al block (T0) and the heat sinks (T1) recorded the variation of the temperatures at the Peltier unit. In a second batch of experiments, we estimated the time required by the bioinks to reach a pre-set temperature after being loaded inside the printhead. Syringes filled with water were tested in one of the following setups: (i) heating water from 22 to 37 °C to emulate the cells loading process in hydrogels, and (ii) cooling water from 37 to 10 °C to study the waiting times for printing hydrogels at low temperatures. 

### 2.4. Hydrogel Preparation

Poloxamer 407 (Pluronic^®^ F127; Sigma-Aldrich, Steinheim, Germany) hydrogels were prepared at 40 wt.% by mixing in cold Milli-Q water at 4 °C, homogenized using a centrifuge and stored overnight at 4 °C to remove air bubbles. Gelatin from porcine skin (type A; 300 bloom; G1890, Sigma-Aldrich, Steinheim, Germany) and sodium alginate (low-viscosity from brown algae; A0682, Sigma-Aldrich, Steinheim, Germany) were dissolved in phosphate buffered saline without salts (PBS-/-, Sigma-Aldrich, Steinheim, Germany) to prepare hydrogels at 20 and 4 wt.%, respectively. Gels were sterilized by autoclave at 120 °C for 20 min, and stored at 4 °C. The pH of the gels was adjusted to 7.2–7.4 prior to use. Solutions were mixed using vortex and centrifuged at 37 °C for 1 min to remove air bubbles.

### 2.5. Rheological Characterization of the Hydrogels

The rheological properties of Gel and Gel–Alg blends were measured using a stress-controlled rheometer (AR-G2; TA Instruments, New Castle, DE, USA) fitted with a temperature-controlled Peltier plate. All tests were performed using a 20 mm parallel plate configuration. Storage modulus (G´) and loss modulus (G´´) were measured at a frequency of 1 Hz and an oscillatory strain of 1%, keeping the blends within the linear viscoelastic region. The equilibrium time required by samples to proceed with the experiment was 5 min, except for the time sweep tests that did not include any equilibrium time. Temperature sweep tests were performed at a rate of 1 °C/min over a range of temperatures from 5 to 45 °C. Time sweep samples were loaded at 37 °C, cooled to the target temperature and tested for 1800 s. The viscosity of the hydrogels was measured under continuous-flow steady state conditions at shear rates ranging from 0.01 to 200 s^−1^.

### 2.6. Benchmark Printing Six Calibration Models with P407 Hydrogels

Six calibration models were designed to quantify the resolution of the device proposed. They were carefully selected from previous publications to have results comparable to those from other research groups. These 3D models were designed using an open-source computer-aided design (CAD) software FreeCAD (v0.17, https://www.freecadweb.org/) [41], and exported as STL files (available under request). A detailed description and justification of the calibration models is included in the Supporting Information. Nozzle trajectories and speeds for printing the six models were generated using the Slic3r software (Slic3r, V3, Italia) [42]. Additional post-processing based on custom-made scripts was necessary to adapt the G-Code to the particular characteristics of the printhead. A detailed description and justification of the calibration models is provided as follows:

- Concentric squares [43]: four concentric empty squares of sides 5, 8, 11, and 14 mm were printed varying the number of layers stacked (1, 2, 4, 8, and 16 layers). Squares were aligned with the XY-axes. Square sides in X-Y directions were measured separately. In the case of concentric squares, and the following two models (circles and multilayer lattice structures), the dimensional errors were calculated as the difference between the dimensions of the extruded model and the values of the CAD model.
- Concentric circles [44]: concentric empty circles of diameters 5, 8, 11, and 14 mm were printed varying the number of layers stacked (1, 2, 4, 8, and 16 layers). Circles involved XY-axes movements at the same time. The diameter of all circles was measured and compared to the model diameter to find the accuracy of the combined XY-axes.
- Multilayer lattice structures [45]: pore size (p), strand diameter (d) and strand spacing (ss) were measured varying the number of layers stacked (2, 4, 8, and 16 layers). Predefined values of p = 1.3 mm, d = 0.2 mm, and ss = 1.5 mm were used. When creating lattice models, pore size, strand diameter, and strand spacing were the main quantitative parameters to define the print resolution.
- Straight filaments [46]: 30 mm long straight filaments with different strand widths were printed aligned with the Y-axis using the same tapered nozzle, but varying the deposition speed from 5 to 16.6 m/s.
- Vertical pillars [47]: Pillars were printed without stacking layers by moving along the Z-axis at the same x-y coordinates until the desired pillar height from 2 to 10 mm was reached. Printing parameters, such as pressure and deposition speed (0.83 to 4.16 mm/s), were adjusted to withstand their vertical shape and avoid the collapse. Stability was evaluated based on the final straightness of the pillars for different heights and the outcome categorized into three categories: (i) stable, if no bending was observed, (ii) unstable, if the pillar bent to one side, and (iii) collapsed, if the post bent utterly touching the glass slide.
- Hierarchical networks of filaments with varying diameter [7]: the printed model simulates the potential creation of a hierarchical vascular network. The connected network of curved filaments was printed in four different sections with the same nozzle size at different speeds to change the printed diameter.

The calibration models presented were printed with 40 wt.% P407 on 25.4 × 76.2 × 1 mm glass slides. The P407 was first loaded into the syringe barrel at 4 °C and extruded setting temperature to 22 °C and pressure to 124 kPa. The deposition speed for the calibration models 1, 2, and 3 was adjusted to 15 mm/s to obtain optimal printing results. The straight filaments were extruded in a range of deposition speeds from 5 to 16.6 mm/s. The vertical pillars have heights ranging from 2 to 10 mm and were generated varying the vertical speed from 4.16 to 0.83 mm/s. Finally, the hierarchical network was extruded with the following printing speeds on each section: S1: 2.5 mm/s; S2: 5 mm/s; S3: 8.3 mm/s; and S4: 15 mm/s. The same tapered nozzle (27G; inner diameter (ID) = 200 μm; Nordson EFD) was utilized in all cases. 

To evaluate the print performance photographs and videos were taken using a Digital Single Lens Reflex (DSLR) camera (Canon EOS 700D, Canon^®^ Inc., Tokyo, Japan). Six samples were created per calibration model (*n* = 6), and each feature was measured at least five times per sample using ImageJ v2.0 software (National Institutes of Health, Bethesda, MD, USA) [48].

### 2.7. Stem Cells Isolation and Expansion

Human adipose-derived mesenchymal stem cells (hASCs) were isolated from lipoaspirate procedures from healthy donors, aged between 18 and 35, following written informed consent and Research Ethical Board approval by Clinica Isabel Moreno and Hospital General Foundation, Valencia, Spain. Donors were previously screened for human immunodeficiency virus (HIV), hepatitis C and other infectious diseases. hASCs were isolated and expanded following the protocol described by Escobedo-Lucea et al. [49] and harvested with Tryple^®^ (Invitrogen, Carlsbad, CA, USA) at 80% confluence. Their undifferentiated stem cell profile was assessed by flow cytometry at the beginning of the experiments. Cells were positive for CD90, CD73, CD29, CD105, CD146, and CD166 and negative for CD34 and CD45 (data not shown).

### 2.8. Cell-Laden Constructs Bioprinted Using Gel–Alg Blends

Cells were mixed with the bioink (cell density of 10^6^ cells/mL) by gentle pipetting to create a homogeneous suspension that was transferred into a 3 mL Luer-lock syringe (Nordson EFD, Nordson EFD Optimum, USA) and closed with a piston (SmoothFlow, Nordson EFD Optimum, USA). The barrel was connected with a software-controlled solenoid valve and an air pressure regulator for precise control of the pressure between 96 and 110 kPa. Extrusion was performed under controlled nitrogen pressure, previously filtered using a 40 µm sterile filter. The syringe with the mixture was loaded into a preheated/precooled printhead for the stabilization of the hydrogel during 30 min. The bioinks were extruded into 3D cell-laden structures (12 mm × 12 mm) of 4 layers on 35 mm Petri dishes through 25G tapered nozzles (Nordson EFD, Nordson EFD Optimum, USA) at a printing speed of 14 mm/s. The 3D printed constructs were finally cross-linked in 3 wt.% calcium chloride (CaCl_2_; Wako, Japan) for 6 min and then washed three times with phosphate buffer (PBS) and replaced with growth medium, Dulbecco’s modified Eagle’s medium (DMEM, Invitrogen, Carlsbad, CA, USA) supplemented with 6% human serum.

### 2.9. Cell Viability Assay

Cell viability in the printed constructs was assessed by live/dead assay (R37601; Life Technologies, Darmstadt, Germany) according to manufacturer’s instructions. Briefly, live green (A) (Calcium-AM; 0.5 μL/mL) and dead red (B) (ethidium homodimer; 2 μL/mL) were prepared in culture media. Cross-linked samples were incubated for 15 min at RT. Fluorescence images of printed samples were captured 1 and 24 h after deposition under a laser scanning confocal microscope (Olympus FV1200, Olympus, Tokyo, Japan). Three independent samples were utilized for the assay (*n* = 3), with seven stacked images per sample (10 layers).

### 2.10. Statistical Analysis

Statistical analysis of results was performed by one-way analysis of variance (ANOVA) and Student *t*-test using R software (version 3.4.3, R Foundation for Statistical Computing, Vienna, Austria) [50]. *P*-values less than 0.05 were considered statistically significant unless otherwise noted. Data are represented as mean ± standard error of the mean (s.e.m.) of six samples (*n* = 6) unless otherwise noted.

## 3. Results

### 3.1. Temperature Limits, Control, and Performance of the Printhead

**Figure 1:**
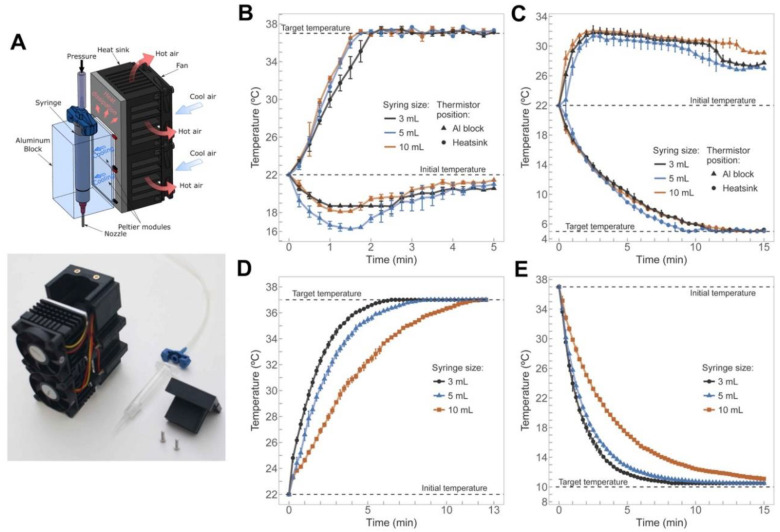
(**A**) Operating principle of the printhead in cooling mode (top) and the printhead assembled and other ancillary components (bottom). (**B**,**C**) Temperature inside the Al block of the printhead and between the heat sinks when: (**B**) heating from 22 to 37 °C, and (**C**) cooling from 22 to 5 °C. (**D**,**E**) Temperature inside the syringe filled with water when: (**D**) heating from 22 to 37 °C, and (**E**) cooling from 22 to 10 °C.

A schematic representation of the operating principle and the printhead assembled with all the ancillary components are shown in Figure 1A. The printhead supports three sizes of syringes, so the maximum and minimum working temperatures of each one was determined for the proper selection of materials and thermoelectric cooling (Peltier) modules (Table S1). When heating, the Al block can rapidly reach temperatures up to 80 °C, but we limited the temperature to 60 °C to avoid deformation of the polypropylene syringe barrels. The design seems suitable for much higher temperatures if we use stainless steel syringes and other printable polymers such as polycarbonate [51]. The maximum time required for heating any of the Al blocks from 22 to 60 °C was only 6 min. On the other hand, reaching the minimum temperatures between 2 and 4 °C required around 15 min. Importantly, temperature and time variations between the different syringe sizes were almost negligible (Table S1), highlighting the appropriateness of the design chosen for 3D bioprinting.

Precise and stable temperature control is essential for maintaining high cell viability when extruding thermoreversible bioinks with high print resolutions [52]. We evaluated the dynamic response of the printhead under heating and cooling cycles. Heating the Al block from 22 to 37 °C did not last more than 2 min with the heat sink temperature only decreasing 6 °C (Figure 1B). On the other hand, cooling down the Al block from 22 to 5 °C spent much more time (around 13 min) (Figure 1C) but the temperature of the heat sinks never exceeded 32 °C. Under these conditions, the variation of the steady-state temperatures was always within ±0.3 °C, an acceptable margin of error in terms of temperature control to avoid cells experience thermal-induced damage.

Under different heating and cooling conditions, we also monitored the temperature inside the three syringes mounted on the printhead and filled with water. The use of water emulates a hydrogel as water mass fraction in hydrogels is significantly high [53]. We found that the time required to increase the water temperature from 22 to 37 °C in the barrel with the largest volume (10 mL) was double than that in the smallest one (3 mL) (Figure 1D). Reducing the water temperature in the syringes from 37 to 10 °C caused similar results with 8 and 15 min for the 3- and 10-mL sizes, respectively (Figure 1E). These values help to understand the thermal inertia of this printhead, showing that there is almost no difference between using 3- or 5-mL sizes. They also provide the additional time required for the stabilization of the bioinks after changing their temperature, especially in the case of working with large volumes (10 mL).

### 3.2. Rheology of the Gel–Alg Bioinks

**Figure 2:**
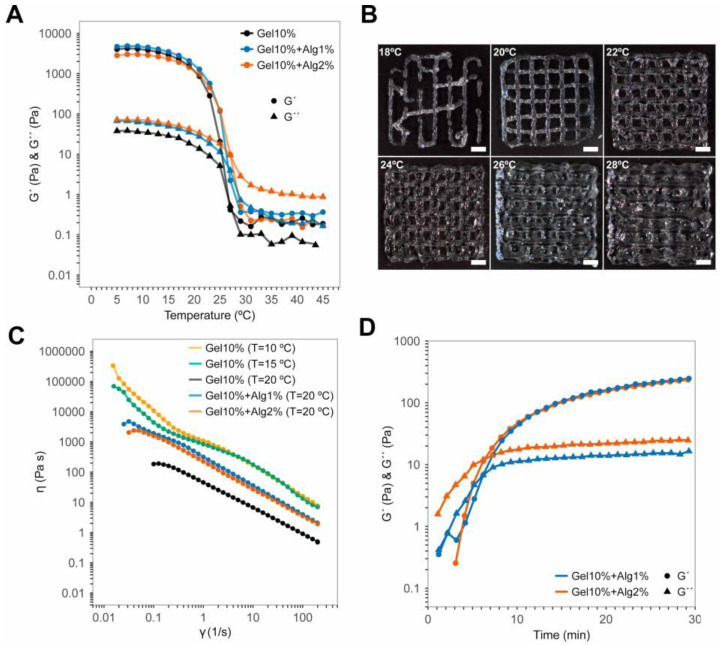
(**A**) Rheological analysis of the gelation points, G´ and G´´ at 20 °C. (**B**) Lattice structures printed in 10%Gel–2%Alg at temperatures ranging from 18 °C (upper-left) to 28 °C (lower-right). Scale bars: 2 mm. (**C**) Viscoelastic behavior of the Gel–Alg bioinks at different temperatures. (**D**) Stabilization times of Gel–Alg blends at 20 °C.

Printability of natural polymer inks is still challenging [54]. The rheological properties of the Gel–Alg blends near the phase transition temperature were determined as an initial step to analyze their printability using our device. As shown in Figure 2A, the gelation point of 10 wt.% Gel was around 26 °C. Below that, gels undergo thermally-reversible gelation. In line with previous works [55], Gel mixed with 1 and 2 wt.% Alg increased slightly the phase transition temperature up to 27 and 28 °C, respectively. The printability of 10% Gel–2% Alg blends was studied by creating a two-layer lattice models at different temperatures. Figure 2B proves the importance of finding the optimal print temperatures to assure high resolution (between 20 and 24 °C for this particular case).

We characterized the shear thinning behavior of the bioinks employed with continuous steady shear tests at 10, 15, and 20 °C. The results showed that the apparent viscosities ranged from 200 to 30,000 Pa·s at a shear rate of 0.1 s^−1^ (Figure 2C). The 10 wt.% Gel hydrogels showed small changes in their viscosities between 10 and 15 °C, but a significant reduction of two orders of magnitude at 20 °C. The Gel–Alg blends exhibited both similar shear thinning behavior at 20 °C with apparent viscosities around 1000 Pa·s at 0.1 s^−1^. This value lies within the range of viscosities of Gel described above. The addition of Alg increased the viscosity at 20 °C by an order of magnitude compared to the Gel alone. Therefore, if other printing conditions remain unchanged, the Gel–Alg blends may decrease cell viability due to their higher viscosity.

A preliminary holding time at the print temperature is required to stabilize the thermoreversible bioinks at the desired rheological properties [56]. The oscillatory time sweep tests at 20 °C showed that the Gel–Alg blends required 30 min for their complete stabilization (Figure 2D). This value was taken as the holding time for all the bioprinting experiments performed with the cells embedded in the Gel–Alg blends.

### 3.3. Print Resolutions Using Three Different 3D Printing Platforms

**Figure 3:**
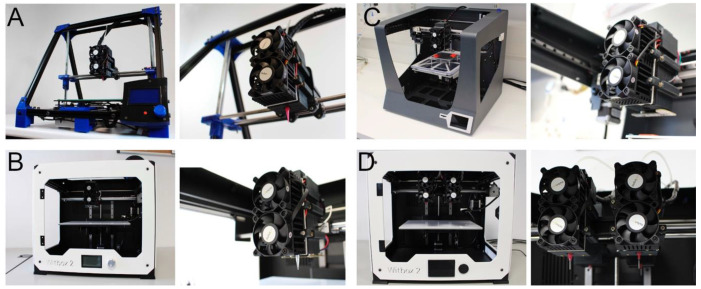
General view and detail of the printhead installed in three open-source desktop 3D printers: (**A**) kit BCN3D+, (**B**) factory-assembled Witbox2, (**C**) factory-assembled Sigma printer, and (**D**) the same Witbox2 with multiple printheads installed on it.

The printhead could be installed easily in three open-source FDM 3D printers, proving its versatility (Figure 3A–C). Multiple printheads were also installed on one of these 3D printers by just redesigning the carriage (Figure 3D), and opening new possibilities for creating multimaterial 3D architectures [57].

**Figure 4:**
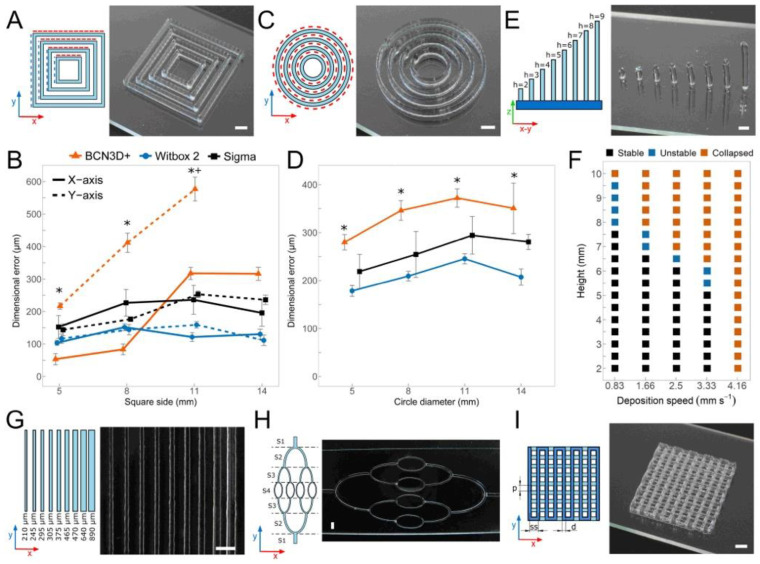
Schematic illustrations and optical images of the 3D calibration models: (**A**) Concentric squares aligned with XY-axes; (**B**) dimensional errors obtained printing concentric squares of eight layers in 40 wt.% P407. Note that asterisks (*) and cross symbols (+) indicate statistical significance (*p* < 0.05) between XY-axes of BCN3D+ and Witbox2, respectively; (**C**) concentric circles; (**D**) dimensional errors obtained printing concentric circles of eight layers. Asterisks (*) indicate statistical significance between BCN3D+ and Witbox2 printers (*p* < 0.05); (**E**) equally distributed pillars with different heights; (**F**) stability of the vertical pillars printed at different deposition speeds; (**G**) parallel filaments of width proportional to deposition speed; (**H**) hierarchical vascular network with filaments of different width; (**I**) multilayered lattice structure. Scale bars: 2 mm.

We evaluated the dimensional errors and the print resolution by generating six types of calibration models in 40 wt.% Poloxamer 407 (P407) with each platform (Figure 4). As aforementioned, the models are mostly based on simple based on previous publications to create constructs comparable to those from other research groups [43,44,58,59,60]. The P407 hydrogel, which rheological properties can be found elsewhere [7], was used because this synthetic polymer has a reduced post-printing swelling [22].

The concentric squares of the first calibration model printed (Figure 4A) had sides of 14, 11, 8, and 5 mm and a different number of stacked layers. We compared the dimensional errors obtained in X and Y axes independently (Figure 4B and Figure S5). We expected that dimensional errors would increase with number of layers stacked for all the bioprinters but the increment was significantly higher (*p* < 0.05) in the BCN3D+ than in the other 3D printers. In addition, there were no differences between the dimensional errors in X and Y axes printing with the Witbox2 or Sigma; whereas the BCN3D+ showed significantly higher (*p* < 0.05) errors in the Y-axis (up to 500 µm for the largest squares) than in the X-axis (around 320 µm for the largest squares). This is an important limitation because it was impossible to create concentric squares of 14 mm side and more than eight layers with this machine. This suggests that the print resolution in the BCN3D+ was very limited due to unforeseen mechanical limitations (Video S1 and S2). Printing squared calibration models of 16 layers was also challenging but both Witbox2 and Sigma 3D printers showed enough positional precision to pass the test (Figure S5). Regardless the number of layers stacked and the square size, the printhead installed in the Witbox2 was the only case with dimensional errors between 41 to 204 µm while Sigma values were between 98 to 263 µm for all the conditions.

Another calibration model (Figure 4C) consisted of four concentric circles with a varying number of layers (Video S3). Circular, cylindrical, and semi-spherical models can be used to evaluate the print resolution of the printhead during the simultaneous movement in both XY-axes. Once again, the errors obtained with the BCN3D+ were always larger than those of other bioprinters (Figure 4D). Witbox2 was again the most accurate bioprinter with errors ranging from 80 to 343 µm, but differences with Sigma were not statistically significant. In contrast to squared-based calibration model, all the bioprinters were capable of creating cylindrical models of up to 16 layers (Figure S6).

The third calibration model consisted in slender pillars (Figure 4E and Video S4) printed at deposition speeds lower than 4.16 mm/s. The stability of the printed posts was inversely proportional to their height and Z-axis speed. The highest stability (Figure 4F) was obtained at minimum deposition speed of 0.83 mm/s with a total height of 7.5 mm. We also created a set of parallel straight filaments with different widths at constant pressure but varying Y-axis speed from 5 to 16.6 mm/s (Figure 4G). Similar filament widths were obtained for the three 3D printers, indicating that these were not dictated by the mechanics of the printers. The MEBB printhead, independently of the printer used, produced threads of width 600 μm at low deposition speeds and slightly above 210 μm at maximum deposition rate (Figure S7). Hierarchical vascular networks of filaments with varying width were also generated in a combined XY-axis movement (Figure 4H and Video S5). In this case, we can consider a priori that the 3D printer mechanics may be involved. Interestingly, all bioprinters managed to produce the desired models without discontinuous elements at constant pressure and within an acceptable range of deposition speeds (3.3–13.3 mm/s).

**Figure 5:**
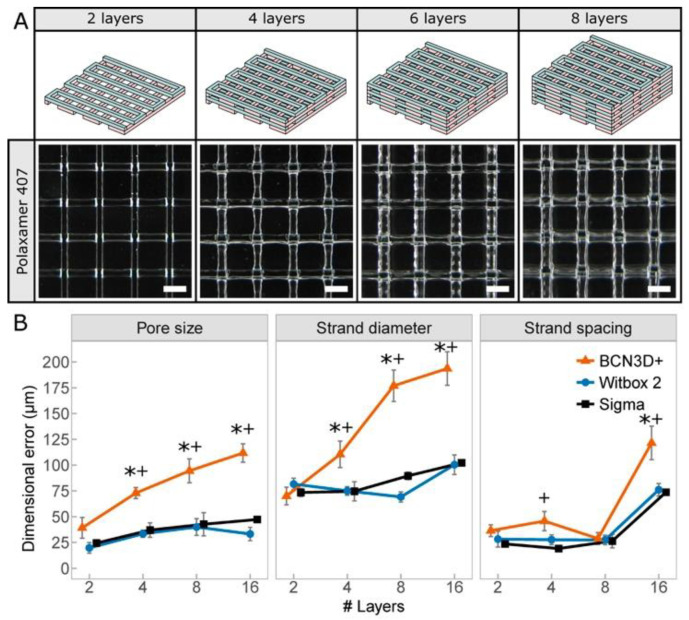
(**A**) Schematic representation (top row) and representative photos of multilayered lattice structures (bottom row) created in 40 wt.% P407 using the printhead installed in the Witbox2. (**B**) Dimensional errors of the printed lattice structures. Asterisk symbols (*) indicate statistical significance between BCN3D+ and Witbox2 (*p* < 0.05). Cross symbols (+) indicates statistical significance between BCN3D+ and Sigma (*p* < 0.05).

Multilayer lattice structures (Figure 4I) are the most common 3D models in bioprinting. We created (Figure 5A and Video S6) several of these structures controlling the main variables: pore size and strand diameter. Both are essential to assure a proper structural stability and high porosity in long-term cell cultures, and therefore, excellent cell viability. Concerning both variables, Witbox2 and Sigma bioprinters showed a similar performance (Figure 5B) and in both cases significantly better than that of BCN3D+. The maximum error in pore size of the Witbox2 (39 ± 8 μm) was almost three times lower than that of the BCN3D+ (111 ± 8 μm). The weak performance of our printhead in the BCN3D+ is particularly significant when printing more than two layers, showing a clear limitation of the system for the creation of 2D patterns. BCN3D+ performance significantly decreased when printing lattice structures of 16 layers (Figure 5B). The errors in the strand spacing did not differ so much between the three MEBB systems.

### 3.4. Bioprinting Cell-Laden Lattice-Shaped Constructs

**Figure 6:**
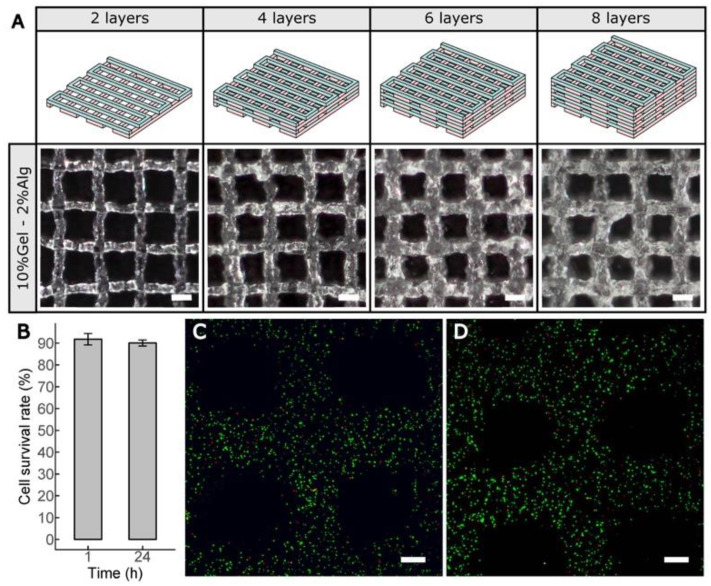
(**A**) Schematic representation (top row) and micrographs (bottom row) of the multilayered cell-laden lattice structures printed in 10%Gel–2%Alg. Scale bars: 1 mm. (**B**) Cell viability after bioprinting hASCs embedded in 10%Gel–2%Alg bioinks at 20 °C using a 25G tapered nozzle. (**C**,**D**) Representative laser confocal images of cell viability assay 1h (**C**) and 24 h (**D**) after printing. Scale bars: 200 µm.

Our printhead mounted on the Witbox2 3D printer was finally selected to create series of cell-laden lattice structures due to the fine print resolution obtained using P407. According to the rheological characterization and the printability studies (Figure 2), the candidate print temperatures were limited between 20 and 24 °C. To find the optimal value for the 10%Gel–2%Alg blends, two-layer lattice models were printed. We found the lowest dimensional errors printing at 20 °C (Table 1), while higher temperature dramatically increased the errors. Note that an increase of 2 °C in the print temperature doubles the filaments width. The viscosity and consistency of 10%Gel–2%Alg bioinks at 20 °C was enough to create lattice models with up to 16 layers. The width of the printed strands increased slightly with the number of layers, reducing the pore size of the constructs, but we still achieved excellent print resolution (Figure 6A and Table S4).

Bioprinting induces shear and extensional stresses in the cells and can lead to cell damage. Besides, it is a time-consuming process with a negative influence on cell viability. After the pre-established 30 min of stabilization time for the 10%Gel–2%Alg bioinks, cell-laden lattice constructs were created with our printhead, cross-linked, and incubated with fresh medium. Cell viability assay showed (Figure 6B) that more than 90% were still intact after 1h (Figure 6C), and after 24 h in culture (no significant differences) (Figure 6D).

## 4. Discussion

Simply looking at the commercially available 3D bioprinters, it is not a surprise that high-end models show exceptional positional precision. However, by using an open-source MEBB printhead, we were able to adapt different desktop 3D printers into flexible low-cost 3D bioprinters with a competitive print resolution when compared to high-end 3D bioprinters. Three low-cost desktop 3D bioprinters (Figure 3A–C) were easily setup using the same MEBB printhead designed. Unlike most of the open-source bioprinters published [61,62], a multi-material 3D bioprinter was also built by installing four units of the printhead without additional modifications (Figure 3D). This required essentially to print another carriage and some basic components, while other open-source multimaterial 3D bioprinter, like the one designed by Lee et al. [63] are far more expensive and complex. We believe our device and the information provided are enough to make users with little expertise in electronics and mechanical systems capable of exchanging an FDM extruder with the new MEBB printhead and create their own 3D bioprinter.

To assess the print resolution of our proposal, we presented a benchmark test that included representative calibration models similar to the constructs printed in recent publications. The data demonstrates that the print resolutions achieved extruding 40 wt.% P407 were very similar to those found in recent publications [43,44,45,46,47]. However, we discovered significant differences in the dimensional errors between the X- and Y-axis when using the modified BCN3D+ printer. The most likely explanation is a combination of small imperfections in the assembling process, and low quality of essential mechanical parts of this model. However, differences in the working principle may be other source of error because the print bead of the BCN3D+ is in continuous movement along the Y-axis (Video S2) while this does not happen to the Witbox2 and Sigma 3D printers, which platforms only moves vertically (Video S1). Geometrical errors are an important limitation when building large and complex 3D constructs. Overall, the printhead installed in the Witbox2 or the Sigma showed better printing performance than that in the BCN3D+. In fact, the accumulation of printing defects using the BCN3D+ often collapsed the thick constructs printed with more than eight layers. We conclude that the 3D printer BCN3D+ introduced more inaccuracies than the Witbox2 and Sigma, which both are assembled at provider´s factories. Therefore, factory-assembled 3D printers are a priori preferred to DIY 3D printer kits. 

The printhead installed in the Witbox2 and Sigma generated parallel thread and hierarchical networks in P407 of different diameters and similar resolution to other publications. This is the case of the integrated tissue-organ printer (ITOP) presented by Kang et al. [15], in which sacrificial structures of P407 were similarly printed to create microchannels. Furthermore, the interconnection of printed vascular networks can be performed using the vertical structures [47,64] showed in Figure 4F to create complex TE constructs with multiple materials [65]. Therefore, our printhead installed on a low-cost 3D desktop printer was not able to reach the positional precision shown by high-end machines but achieved a reasonable print resolution that could match most of the requirements of many TE laboratories at reduced cost. 

We mentioned that this MEBB printhead might be a flexible alternative device to the use of a commercial system in the market. This is exemplified in its modular design that allows loading syringe of three different volumes, while other high-end printers lack of this feature or require additional modifications. For instance, the use of 10-mL barrels was essential for fast printing multiple series of calibration models without reloading, while creating cell-laden constructs with high cell density was facilitated by using loading the hASCs in the 3-mL syringe barrel. This capability was also seen in another MEBB printhead proposed by Reid et al. [66], however, their device was intended to use for single cell 2D bioprinting without temperature control. 

The low-cost extruder proposed by Roehm and Madihally [67] to evaluate the printability of chitosan–gelatin blends was designed with no control of the temperature in the syringe. However, to handle a range of thermosensitive hydrogels similar to that of high-end 3D printers, the deposition temperature needs to be controlled. In our printhead, we obtained a remarkably wide range of working temperatures from 2 to 60 °C. Also, the use of different syringe sizes did not alter significantly the maximum heating/cooling response times (18 min), which were still lower than the stabilization time of the gelatin or Alg-Gel blends (Figure 2D). Neither the thermal conductivity of syringe nor the existence of a small gap between the syringe barrel and the Al block apparently decreased the heating/cooling performance of the printhead. However, the use of other materials with different water content or lower volumes of the Alg-Gel bioinks could change the values shown in Figure 1.

The deposition process of MEBB systems usually occurs at low pressure and 20–25 °C to reduce the potential cell damage. We highlighted the great cell viability achieved dispensing cells within thermoresponsive bioinks such as the Gel–Alg selected [68]. However, as shown in Figure 2B, temperature control of these bioinks during bioprinting is crucial not to compromise the final resolution [56]. While the Gel–Alg blends remained near the phase transition temperature, small fluctuations in their temperature revealed changes in the storage and the loss moduli of the blends. Thus, without ruling out other parameters such as extrusion pressure, deposition speed or nozzle diameter [19], it seems that a stable and precise temperature in the Gel–Alg blends was a critical factor to produce significant improvements in the print quality of the lattice structures. We also anticipate that the same effect will be displayed in other biocompatible thermosensitive bioinks such as low melting agarose [25], GelMA [69], or even collagen [70].

Once a fine print resolution was achieved as a result of precise temperature control of the Gel–Alg blends, we showed that this adjustment would not result in a significant decrease of cell viability due to the generation of excessive shear stress during the bioprinting process [12]. As shown in Figure 6B–D, hASCs viability for Gel–Alg printed lattices was higher than 90%, that, together with the printability results at different temperatures (Figure 2B), confirm the proper setup of the MEBB system. 

Finally, the designs and specifications for building the printhead are publicly available, reducing the time and labor required to get the MEBB system ready. In contrast to exceptional works like the platform presented by Shim et al. [71], subscribing the design of the printhead to the open-source philosophy is essential for the future of our device. Attractive possibilities are opened up for studies in multinozzle design, embedded extrusion, among others [72]. Overall, the modularity, wide range of printing temperatures and publicly available documentation make our easy-to-build printhead proposed an excellent tool for bioprinting, becoming the first open-source printhead that provides all these capabilities altogether. Summarizing, the open source philosophy can be exported to create low-cost, open, and versatile 3D bioprinters, accelerating the innovation in the field and their further integration into other areas of TE. This framework could help in engaging the next coming generation with the intensive research on tissue engineering.

## 5. Conclusions

We present an open-source printhead for MEBB created to work with thermosensitive hydrogels. Our printhead is an alternative to the use of commercial bioprinters with a modular design that allows the use of different syringe sizes (3, 5, and 10 mL). Similar to other systems, the print temperature can be precisely controlled from 2 to 60 °C, allowing the use of a broad range of bioinks with different viscosities. However, it is advantageous over other solutions because it can be installed in the majority of affordable open-source 3D printers. We showed this versatility by easily installing the printhead in three different open-source 3D FDM printers. 

The print resolution showed creating the calibration models in P407 could be enough for working on many TE applications, even if positional precision of the 3D printers used are a priori lower than other high-priced machines in the market. The lattice structures of Gel–Alg created at different temperatures demonstrated the capabilities of our printhead for controlling the printing conditions related with the rheology of each bioink. Cell viability in the Gel–Alg structures was higher than 90%. With a reduced total cost (lower than US$ 70), the open-source nature of our device guarantees future modifications, and the possibility to expand its use for multimaterial bioprinting. We strongly believe that this printhead represents a significant leap in bringing 3D MEBB to TE laboratories worldwide.

## Figures and Tables

**Table 1:** Dimensions of the CAD models and the multilayered lattice structures printed in 10% Gel–2%Alg at 20, 22, and 24 °C.

Type	Temperature (°C)	Pore Size (mm)	Strand Width (mm)	Strand Spacing (mm)
CAD model	-	1.75	0.25	2
Printed model	20	1.5 ± 0.02	0.4 ± 0.01	1.99 ± 0.02
	22	1.05 ± 0.02	0.82 ± 0.01	1.99 ± 0.01
	24	0.99 ± 0.02	1 ± 0.01	1.99 ± 0.01

## Supplementary Materials

The following are available online at https://www.mdpi.com/2073-4360/12/10/2346/s1, Figure S1: Designed MEBB printhead, Figure S2: Modular design of the printhead, Figure S3: General view of the main parts that compose the printhead proposed, Figure S4: Wiring diagrams of open-source electronics RAMPS 1.4 and Rumba boards, Figure S5: Dimensional errors measured from concentric squares printed in 40 wt.% P407 at room temperature with 1, 2, 4, and 16 layers stacked using three different 3D printers, Figure S6: Dimensional errors measured from concentric circles printed in 40 wt.% P407 at room temperatures with 1, 2, 4, and 16 layers stacked, Figure S7: Width of the parallel straight filaments printed at constant pressure and deposition speed ranging from 5 to 16.6 mm s^−1^. Table S1: List of current commercially available open-source desktop 3D printers, Table S2: Bill of materials of the printhead including quantity, description, provider, and cost, Table S3: Operating times and temperature limits measured in the Aluminum (Al) block under cooling and heating conditions, Table S4. Dimensions of the CAD models and the multilayered lattice structures printed in 10% Gel–2%Alg at 20 °C. Video S1: Witbox2 bioprinter generating squared calibration models, Video S2: BCN3D+ bioprinter generating squared calibration models, Video S3: Concentric circles generated as calibration models, Video S4: Printing slender pillars with the Witbox2 system, Video S5: Witbox2 creating a layer of hierarchical vascular networks over a slide, Video S6: Multilayer lattice structures generated with calibrated bioprinter.

## Abbreviations

3D	three-dimensional
TE	tissue engineering
MEBB	microextrusion-based bioprinting
DIY	do-it-yourself
Al	Aluminum
STL	stereolithography
ABS	acrylonitrile butadiene styrene
P407	Poloxamer 407
PBS	phosphate buffered saline
CAD	computer-aided design
ID	inner diameter
hASCs	human adipose-derived mesenchymal stem cells
HIV	human immunodeficiency virus
DMEM	Dulbecco’s modified Eagle’s medium
ANOVA	one-way analysis of variance
