# Supplementary material for: A Versatile Open-Source Printhead for Low-Cost 3D Microextrusion-Based Bioprinting
Source: Polymers (Basel). 2020 Oct 13;12(10):2346. doi: 10.3390/polym12102346 (PMC7602012; doi:10.3390/polym12102346)
Supplement: Supplementary file 1 [file polymers-12-02346-s001.zip › polymers-972570-supplementary/Supporting information for publication.docx]

**Supporting Information**

A versatile open-source printhead for low-cost 3D microextrusion-based bioprinting

Andres Sanz-Garcia ^1,2,†^, Enrique Sodupe-Ortega ^1,2,3,†^, Alpha Pernía-Espinoza ^3^, Tatsuya Shimizu ^2^ and Carmen Escobedo-Lucea ^1,2,^*

^1^ Division of Pharmaceutical Biosciences. University of Helsinki. Viikinkaari 5 E (P.O. Box 56), 00014, Helsinki, Finland; andres.sanz-garcia@helsinki.fi (A.S.-G.); carmen.escobedo-lucea@helsinki.fi (C.E.-L.)

^2^ Institute of Advanced Biomedical Engineering and Science, Tokyo Women's Medical University, 8-1 Kawada-cho, Shinjuku-ku, 162-8666, Tokyo, Japan; shimizu.tatsuya@twmu.ac.jp

^3^ Department of Mechanical Engineering, University of La Rioja. San José de Calasanz 31, Edificio Departamental, 26004 Logroño, Spain; enrique.sodupeo@unirioja.es (E.S.-O.); alpha.pernia@unirioja.es (A.P.-E.)

† These authors contributed equally to this work.

***** Correspondence: carmen.escobedo-lucea@helsinki.fi

Number of pages: 13

Number of figures: 07

Number of tables: 04

**TABLE OF CONTENTS**

1. Table S1..................................................................................................................S3

2. Table S2..................................................................................................................S4

3. Table S3..................................................................................................................S5

4. Table S4..................................................................................................................S6

5. Figure S1.................................................................................................................S7

6. Figure S2.................................................................................................................S8

7. Figure S3.................................................................................................................S9

8. Figure S4...............................................................................................................S10

9. Figure S5...............................................................................................................S11

10. Figure S6.............................................................................................................S12

11. Figure S7.............................................................................................................S13

**Table S1.** List of current commercially available open-source desktop 3D printers

| 3D Printer | Provider | Dimensions L-W-H (mm) | Print volume X-Y-Z (mm) | Print resolution* X-Y-Z (µm) | Cost (USD) |
| --- | --- | --- | --- | --- | --- |
| Anet A6 | Shenzhen Anet Technology Co | 480-400-450 | 220-220-250 | 12-12-5* | 180 |
| Anet A8 | Shenzhen Anet Technology Co | 500-400-450 | 220-220-240 | 12-12-5* | 220 |
| BCN3D+ | BCN3D Tech. | 480-480-455 | 252-200-200 | 50-50-100 | 1 055 |
| Bukito | Deezmaker | 330-343-254 | 125-150-125 | 50-50-100 | 900 |
| Creality3D CR-10 | Shenzhen Creality 3D Technology | 290-540-650 | 300-300-400 | 12-12-5* | 350 |
| Creality CR-10 Mini | Shenzhen Creality 3D Technology | 400-425-500 | 300-220-300 | 12-12-5* | 300 |
| Fab@Home m1 | DIY | 470-410-460 | 200-200-200 | 15-15-15 | 2 290 |
| Fab@Home m2 | DIY | 482-386-368 | 200-200-200 | 6-3.5-3.6* | 1 760 |
| Hephestos 2 | BQ | 450-605-661 | 210-297-220 | Up to 50 | 1 059 |
| Lulzbot Mini 2 | Luzbot | 457-339-607 | 160-160-180 | Up to 50 | 1 250 |
| Printrbot Simple | Printrbot | 558-533-228 | 200-150-200 | Up to 50 | 509 |
| Prusa i3 Mk3 | Prusa Research | 420-420-380 | 250-210-200 | 10-10-50 | 800 |
| Rostock Max v3 | SeeMeCNC | 430-410-910 | 275(diam)-385 | 50-50-50 | 1 000 |
| Sigma | BCN3D Tech. | 465-440-680 | 210-297-210 | 12.5-12.5-1* | 2 814 |
| Tronxy X5S | Shenzhen Tronxy Technology Co | 658-630-639 | 330-330-400 | 12-12-4* | 305 |
| Ultimaker Original+ | Ultimaker | 342-560-650 | 210-210-205 | 12.5-12.5-5* | 1 055 |
| Ultimaker 2+ | Ultimaker | 342-357-388 | 223-223-205 | 12.5-12.5-5* | 2 009 |
| Witbox2 | BQ | 508-393-461 | 297-210 -200 | Up to 20 | 1 791 |

* In these cases, the print resolution is defined by the manufacturer as the positioning accuracy.

**Table S2.** Bill of materials of the printhead including quantity, description, provider, and cost

| Part Name | Quantity | Description | Provider | Cost (USD) ^b^ |
| --- | --- | --- | --- | --- |
| Printhead carcass | 1 | 3D printed | - | 1.71 |
| Syringe cover | 1 | 3D printed | - | 0.39 |
| Heatsink clamp | 1 | 3D printed | - | 0.06 |
| Heatsink clamp thin | 1 | 3D printed | - | 0.05 |
| 3D printer coupling | 1 | 3D printed | - | 0.68 |
| Aluminum block | 1 | SMC^a^ | Misumi | 13.49 |
| Aluminum plate | 1 | SMC | Misumi | 4.77 |
| M3 brass insert | 6 | SMC | RS Online | 1.52 |
| Thermistor NTC 100k | 3 | SMC | RS Online | 4.68 |
| M3x10 screw | 10 | SMC | RS Online | 4.77 |
| M3x25 screw | 4 | SMC | RS Online | 1.92 |
| Peltier module | 2 | SMC | Hebei I.T. | 14.80 |
| Heatsink | 2 | SMC | StarTech | 17.59 |
| **Total** |  |  |  | **66.43** |
| ^a^ SMC = Standard Mechanical Component; ^b^ Estimated price on the given publication day. | | | | |

**Table S3.** Operating times and temperature limits measured in the Aluminum (Al) block under cooling and heating conditions.

| Operation mode | Syringe volume (mL) | Final temperature (ºC) | Total time (s) |
| --- | --- | --- | --- |
| Cooling | 3 | 3.86 | 900 |
| Cooling | 5 | 2.86 | 900 |
| Cooling | 10 | 4.4 | 1 080 |
| Heating | 3 | > 60* | 363 |
| Heating | 5 | > 60* | 262 |
| Heating | 10 | > 60* | 343 |

* Maximum temperatures were established for the safety of the printed device.

**Table S4.** Dimensions of the CAD models and the multilayered lattice structures printed in 10% Gel-2%Alg at 20 ºC.

| Type | # Layers of printed models | Pore size [p] (mm) | Strand width [d] (mm) | Strand spacing [ss] (mm) |
| --- | --- | --- | --- | --- |
| CAD model | - | 1.75 | 0.25 | 2 |
| Printed model | 2 | 1.5 ± 0.01 | 0.41 ± 0.01 | 1.95 ± 0.01 |
|  | 4 | 1.41 ± 0.02 | 0.5 ± 0.02 | 1.97 ± 0.03 |
|  | 8 | 1.32 ± 0.02 | 0.58 ± 0.01 | 1.92 ± 0.01 |
|  | 16 | 1.31 ± 0.02 | 0.61 ± 0.02 | 1.89 ± 0.01 |


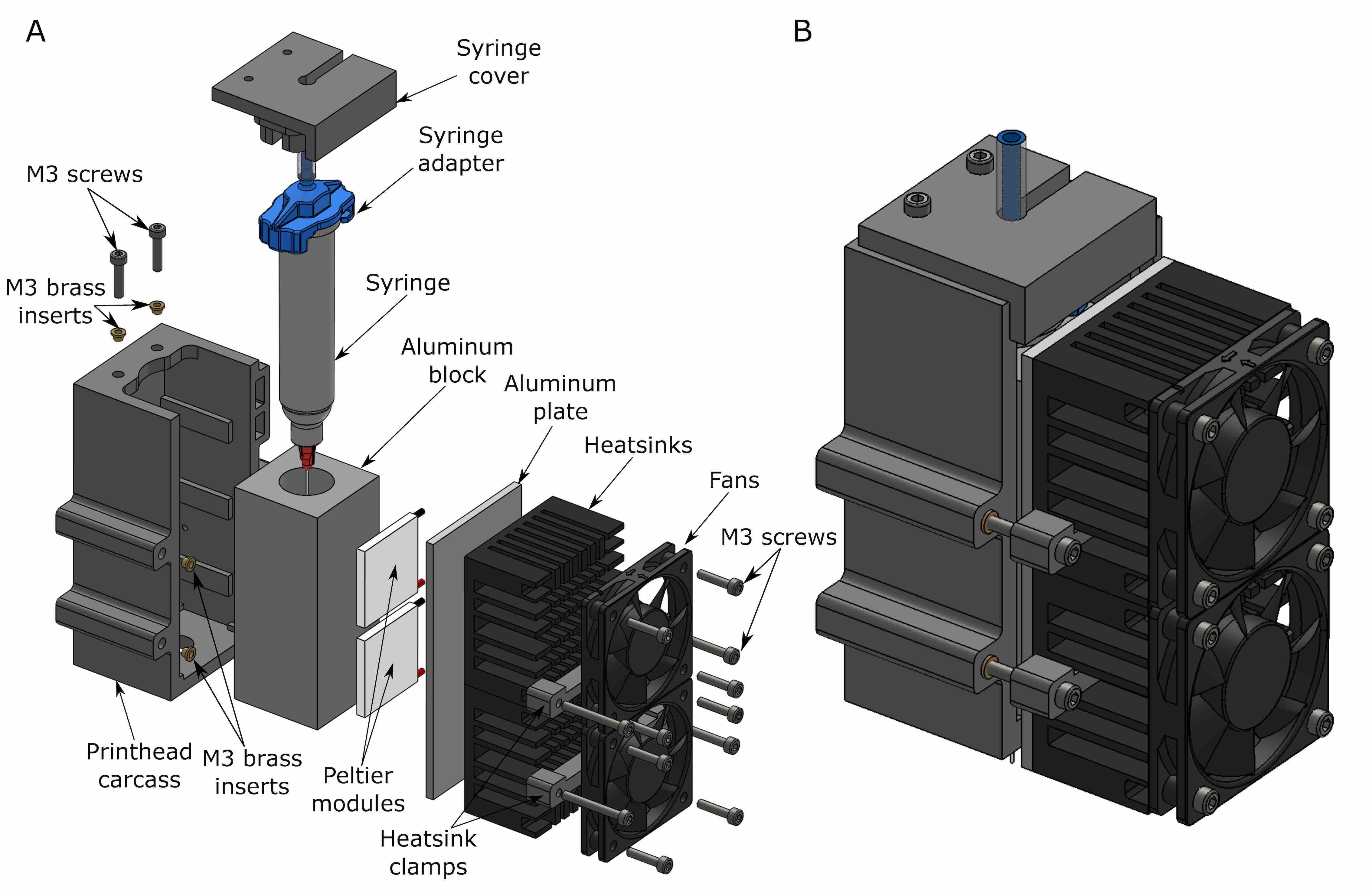


**Figure S1.** (A) Exploded view of the designed MEBB printhead with the schematic presentation of the position of all the components that composed the printhead and the syringe. (B) Schematic representation of the assembled printhead.


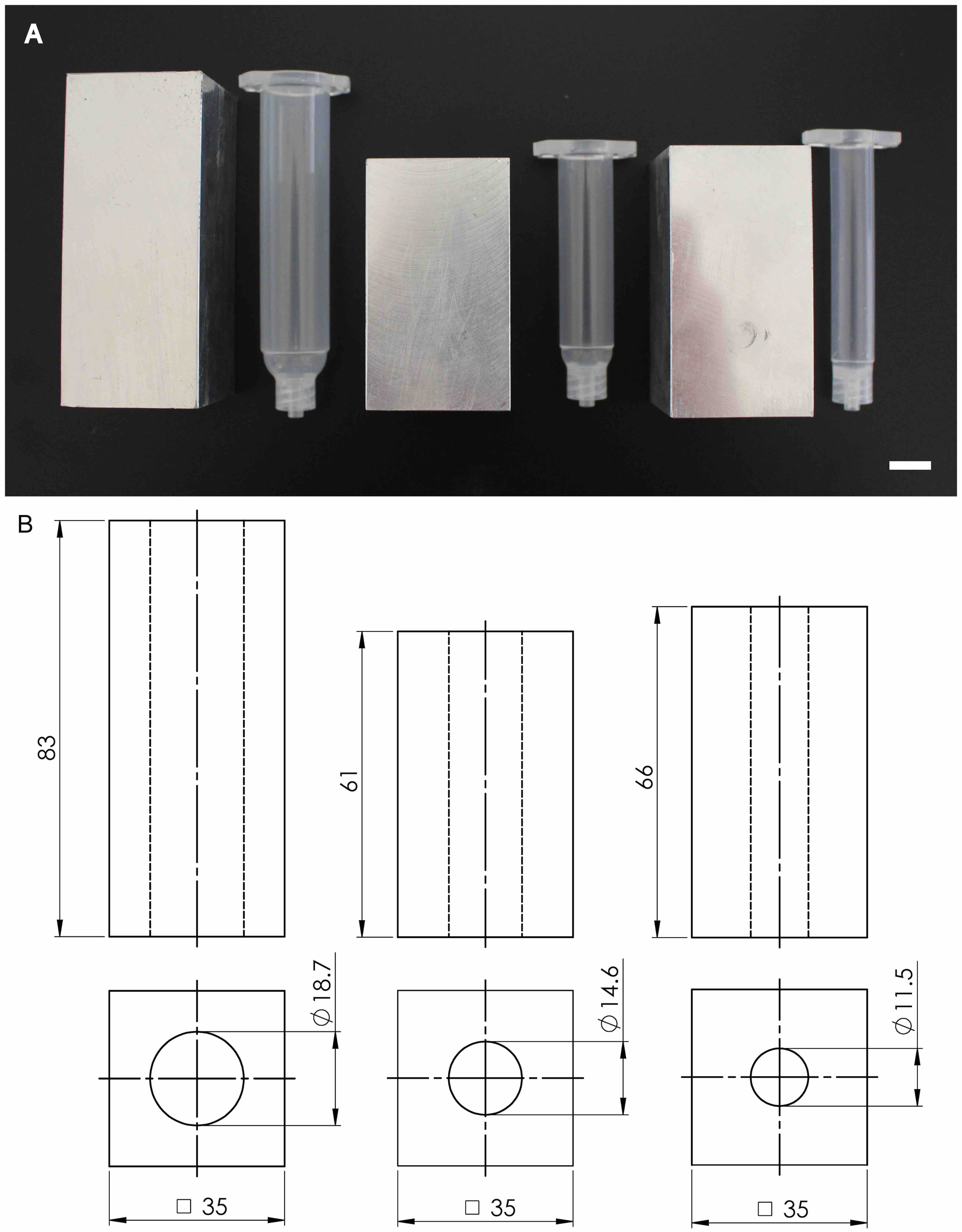


**Figure S2.** Modular design of the printhead. (A) Comparative view of the syringes of 10 mL, 5 mL, and 3 mL (from left to right) with their corresponding Al blocks that provide thermal inertia to the system. Scale bar 10 mm. (B) Dimensioned drawing of the Al blocks for the 10 mL, 5 mL, and 3 mL (from left to right).


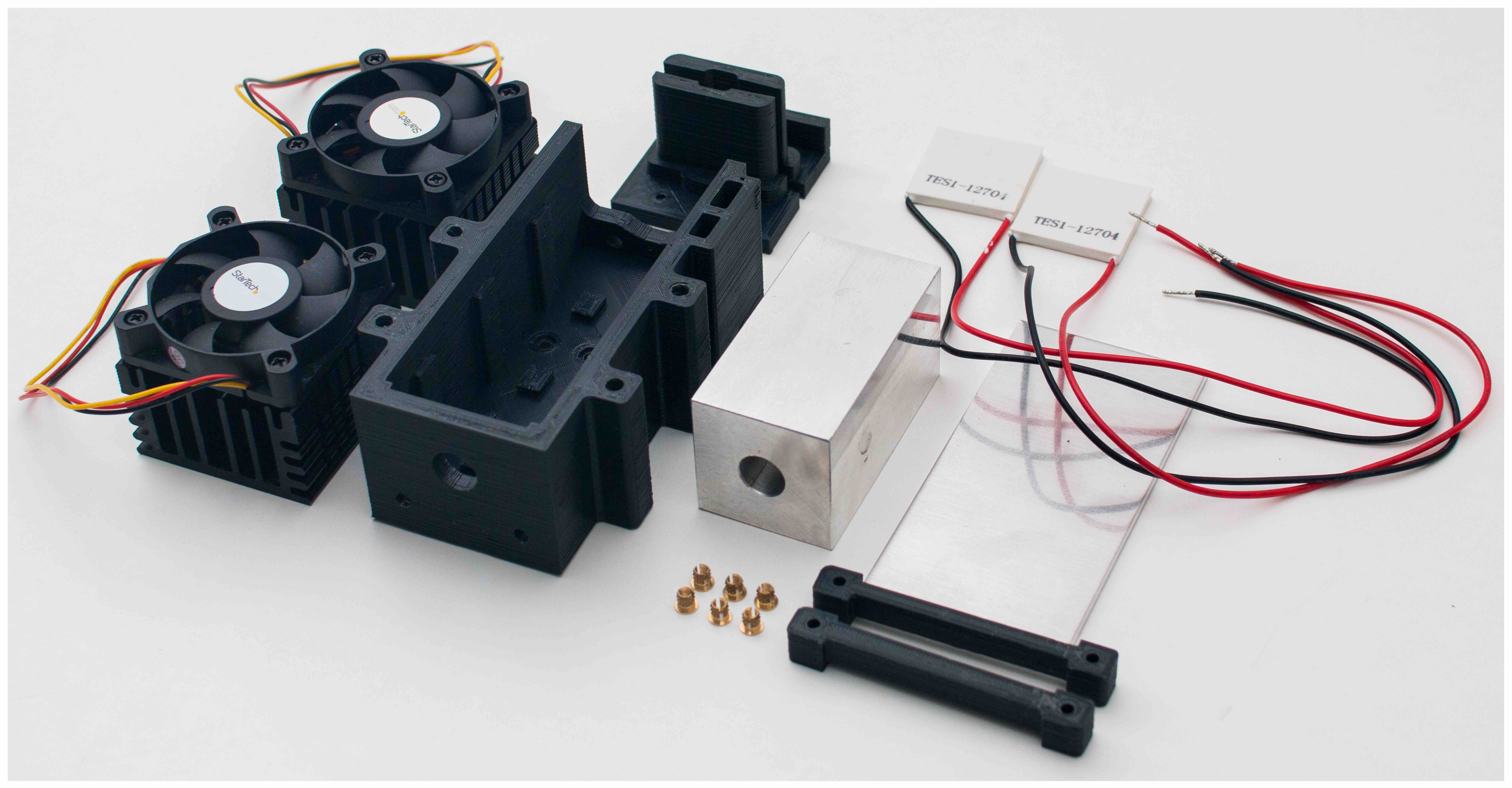


**Figure S3.** General view of the main parts that compose the printhead proposed.


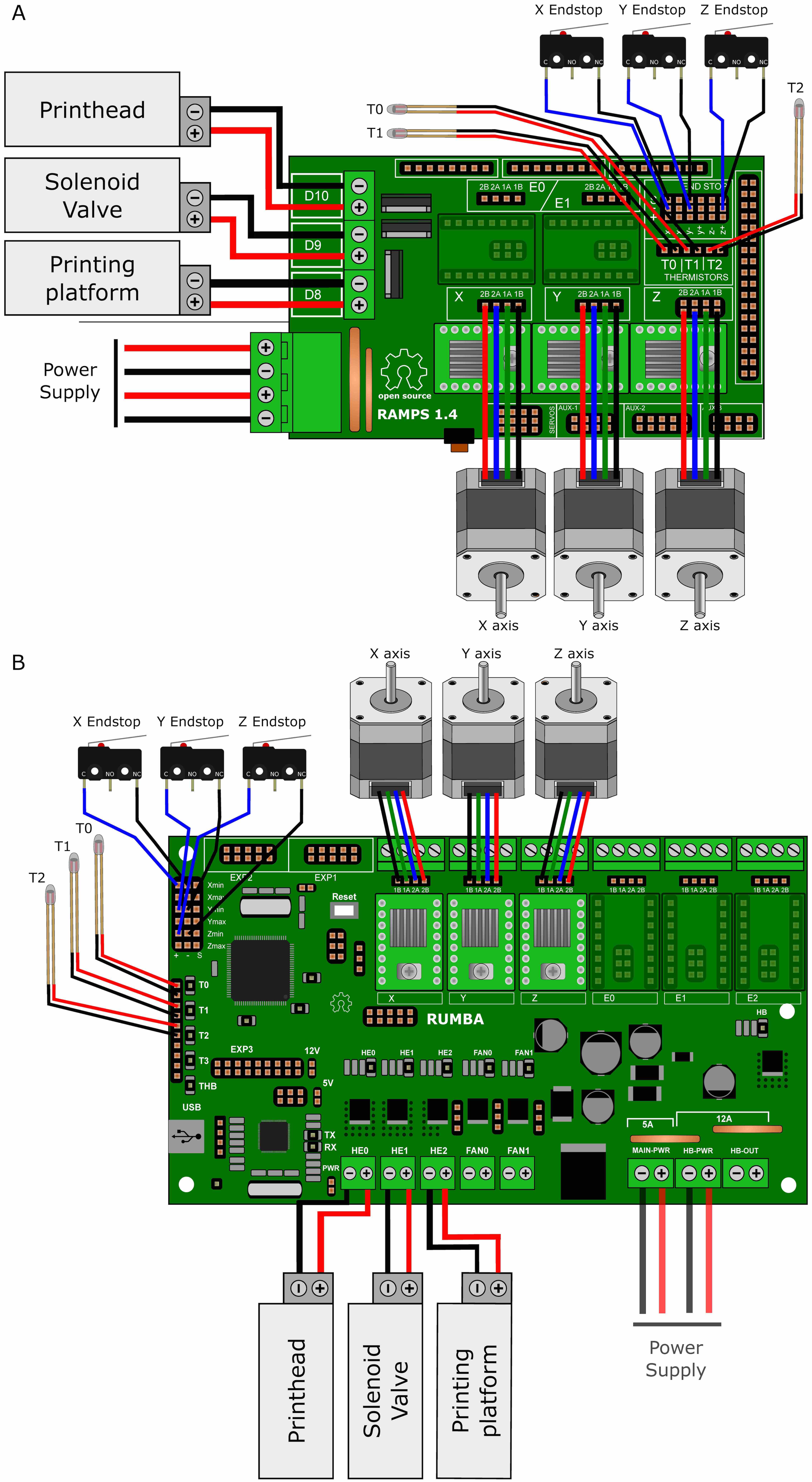


**Figure S4.** Wiring diagrams of open-source electronics RAMPS 1.4 and Rumba boards. Terminals D9, D10 and D8 for RAMPS 1.4 and HE1, HE0 and HE2 for Rumba were used to connect the Peltiers on the printhead, printing platform and solenoid valve. Three thermistors EPCOS 100K were connected to T0 (printhead Al block), T1 (printhead heatsink) and T2 (RT or printing platform) labeled pins. Mechanical end-stops of XYZ-axes were connected to the end-stop board pins as detailed in the figure.


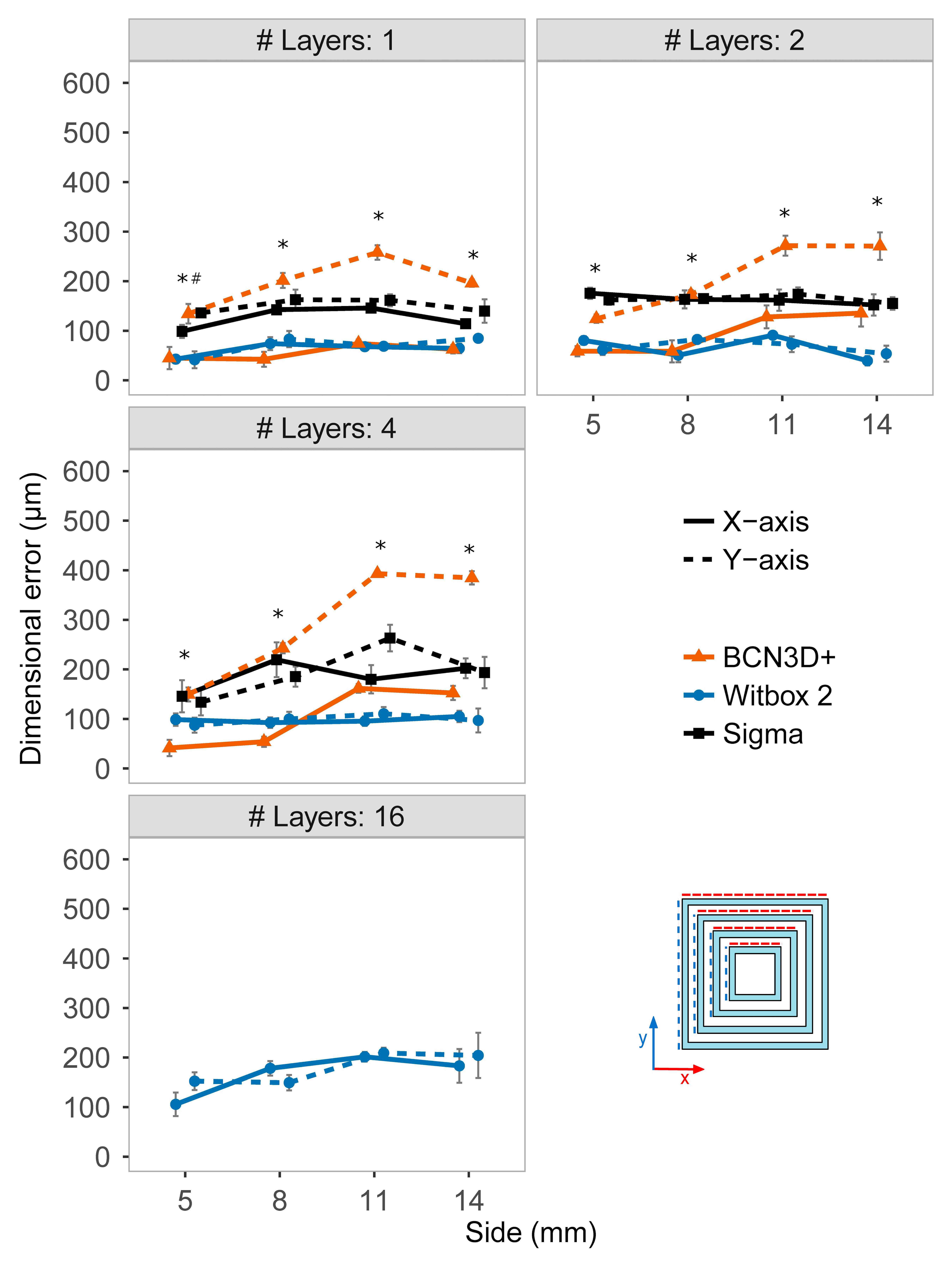


**Figure S5.** Dimensional errors measured from concentric squares printed in 40 wt% P407 at room temperature with 1, 2, 4, and 16 layers stacked using three different 3D printers. Asterisk symbol (*) indicate statistical significance between XY-axes of BCN3D+ (p <0.05), and hash symbols (#) indicate statistical significance between XY-axes of Sigma (p <0.05).


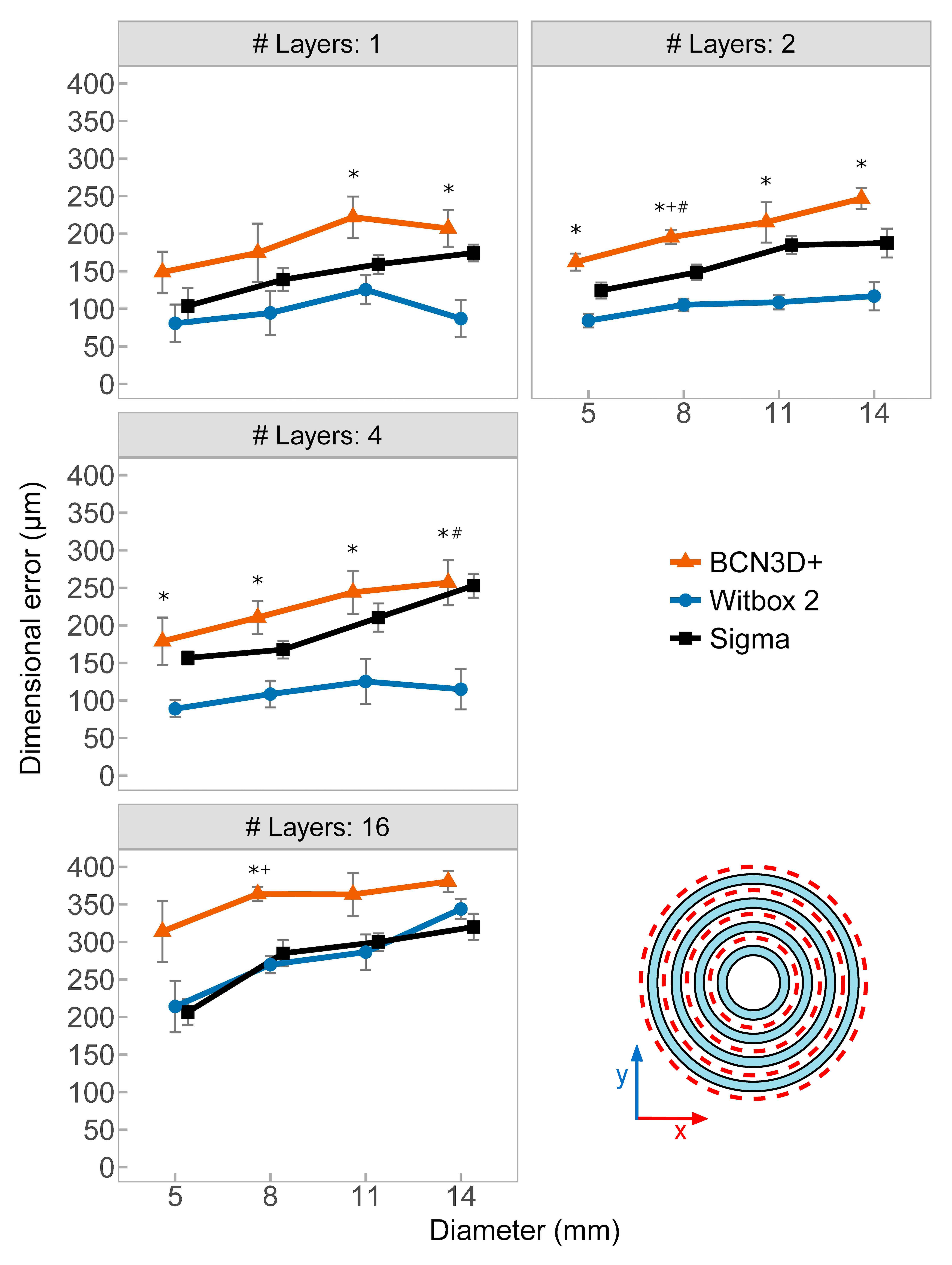


**Figure S6.** Dimensional errors measured from concentric circles printed in 40 wt% P407 at room temperatures with 1, 2, 4, and 16 layers stacked. Asterisk symbols (*) indicate statistical significance between BCN3D+ and Witbox2 (p <0.05). Cross symbol (+) indicate statistical significance between BCN3D+ and Sigma (p <0.05). Hash symbols (#) indicate statistical significance between Sigma and Witbox2 (p <0.05).


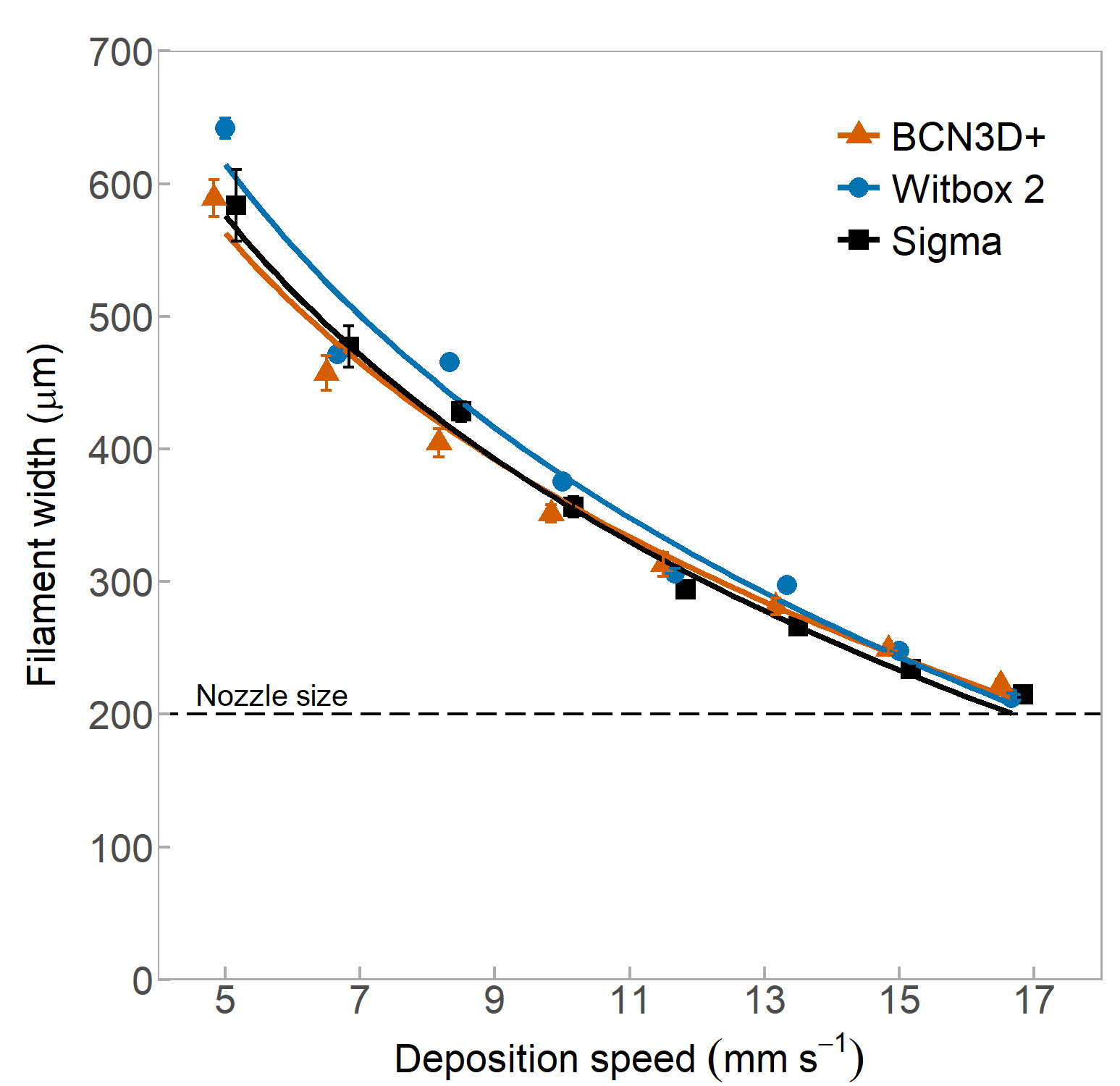


Figure S7. (A) Width of the parallel straight filaments printed at constant pressure and deposition speed ranging from 5 to 16.6 mm s-1. Vertical and horizontal straight filaments were printed in 40 wt% P407 using a 27G tapered nozzle.
