# Supplementary figures and images for: A Versatile Open-Source Printhead for Low-Cost 3D Microextrusion-Based Bioprinting
Source: Polymers (Basel). 2020 Oct 13;12(10):2346. doi: 10.3390/polym12102346 (PMC7602012; doi:10.3390/polym12102346)

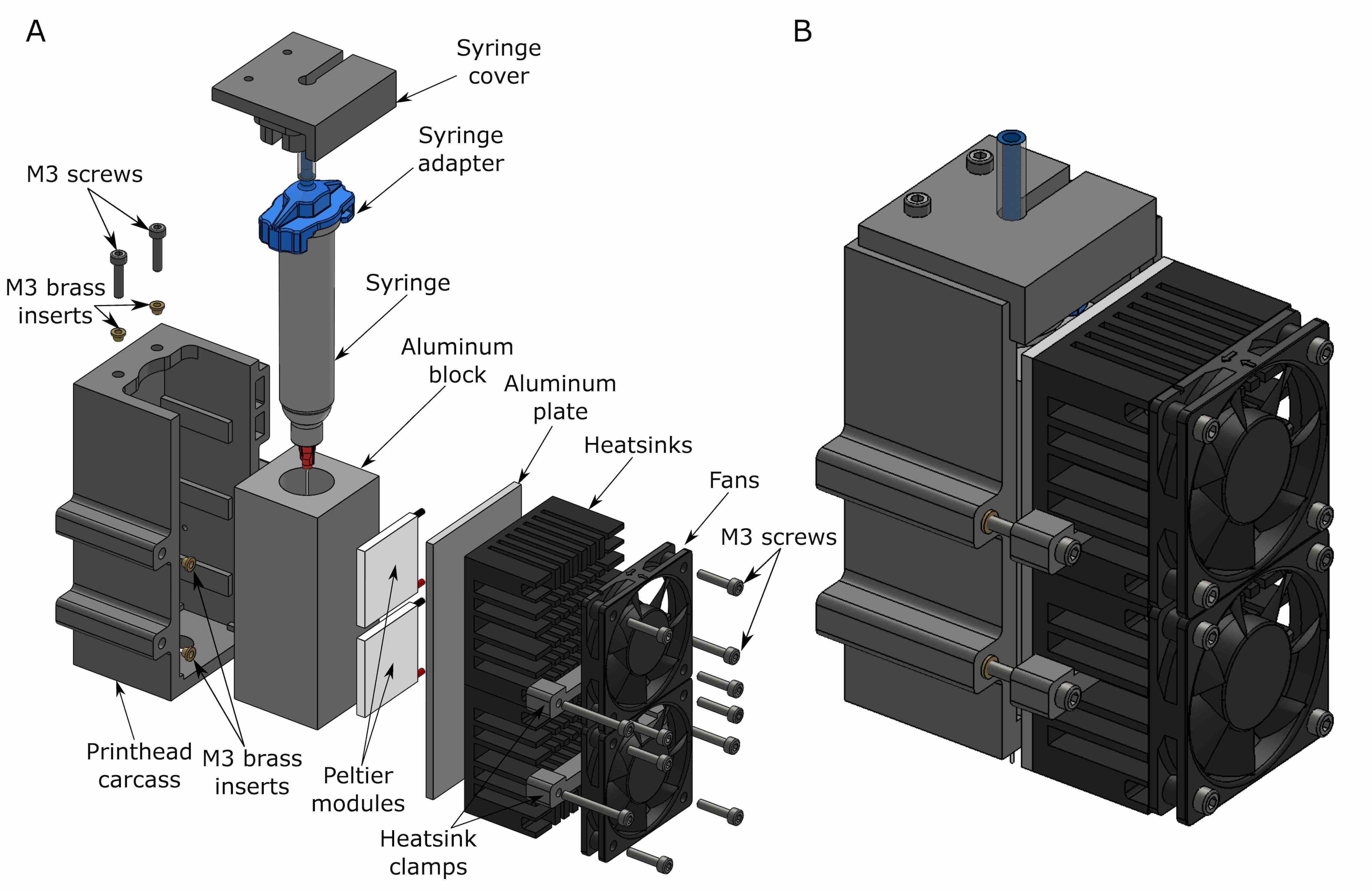

Supplement: Supplementary file 1 [file polymers-12-02346-s001.zip › polymers-972570-supplementary/Supporting Info/figS1.jpg]

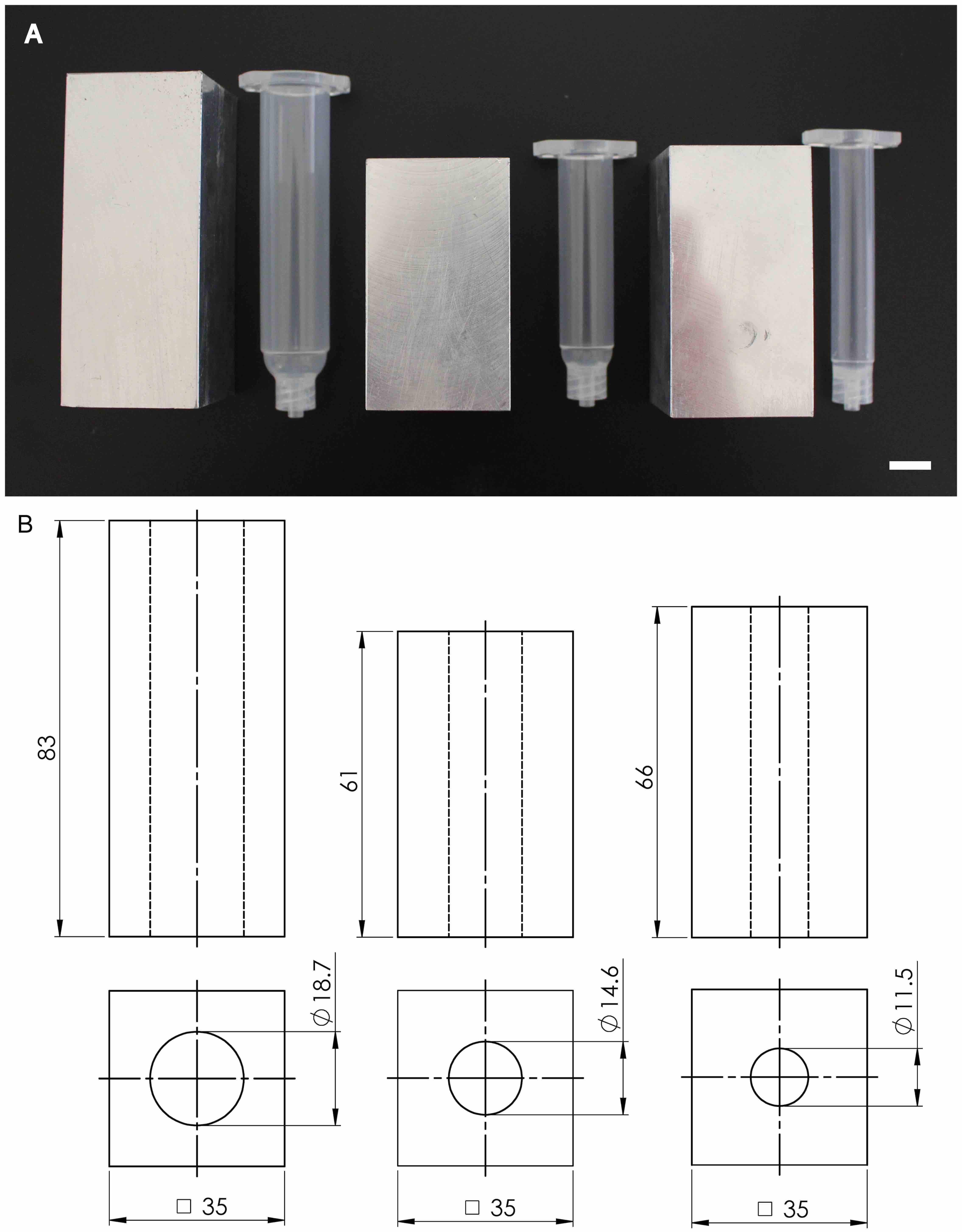

Supplement: Supplementary file 1 [file polymers-12-02346-s001.zip › polymers-972570-supplementary/Supporting Info/figS2.jpg]

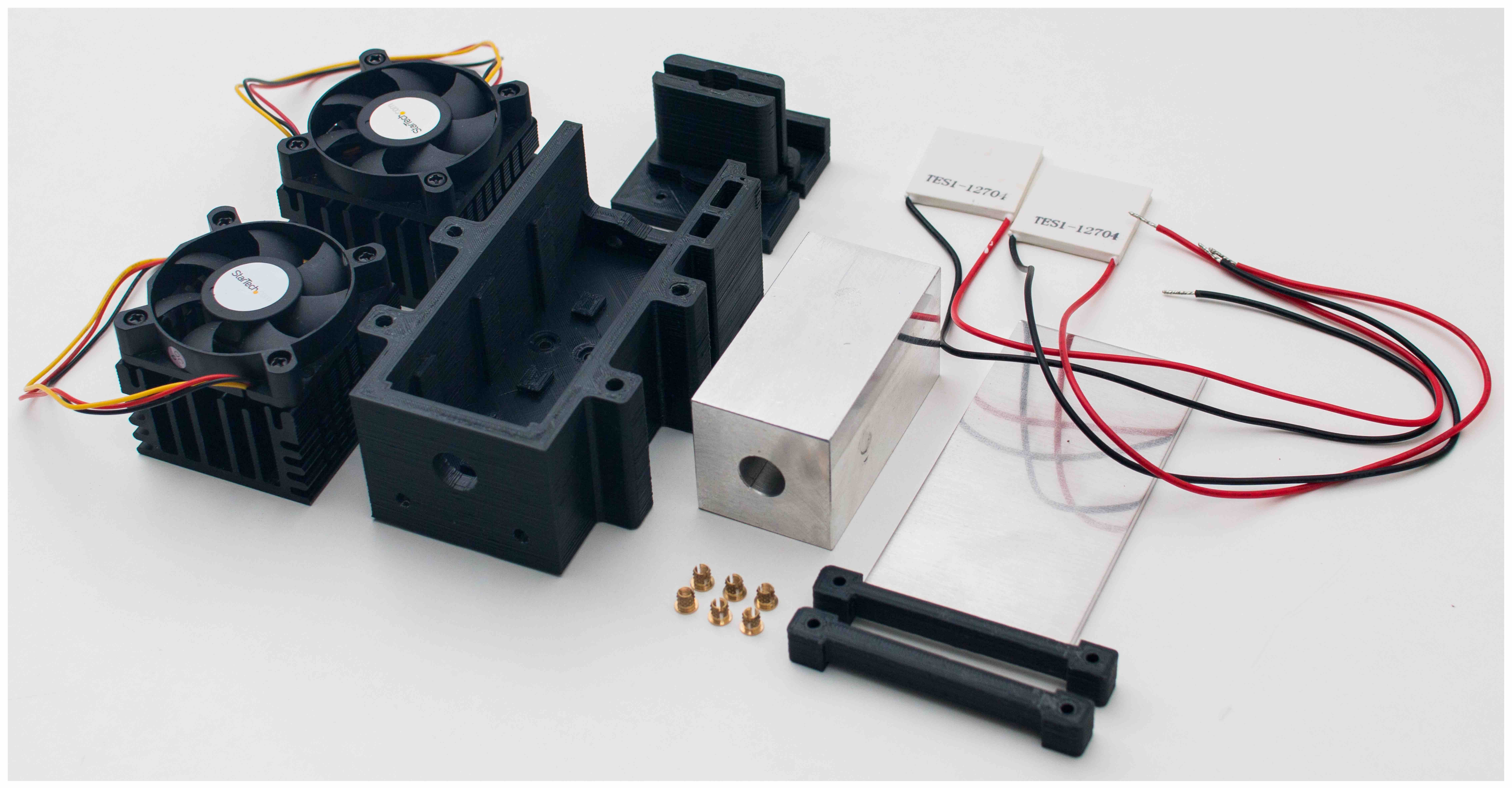

Supplement: Supplementary file 1 [file polymers-12-02346-s001.zip › polymers-972570-supplementary/Supporting Info/figS3.jpg]

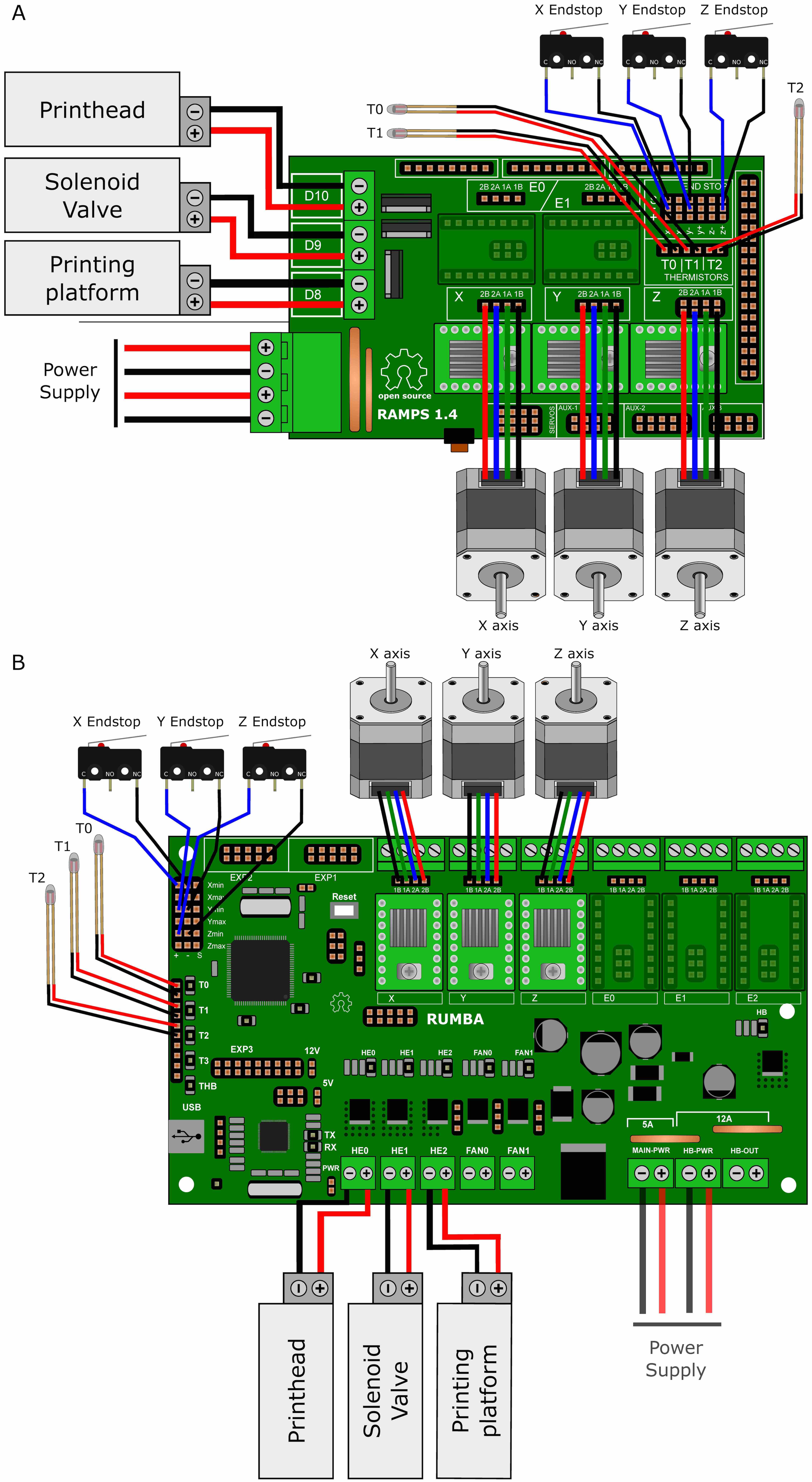

Supplement: Supplementary file 1 [file polymers-12-02346-s001.zip › polymers-972570-supplementary/Supporting Info/figS4.jpg]

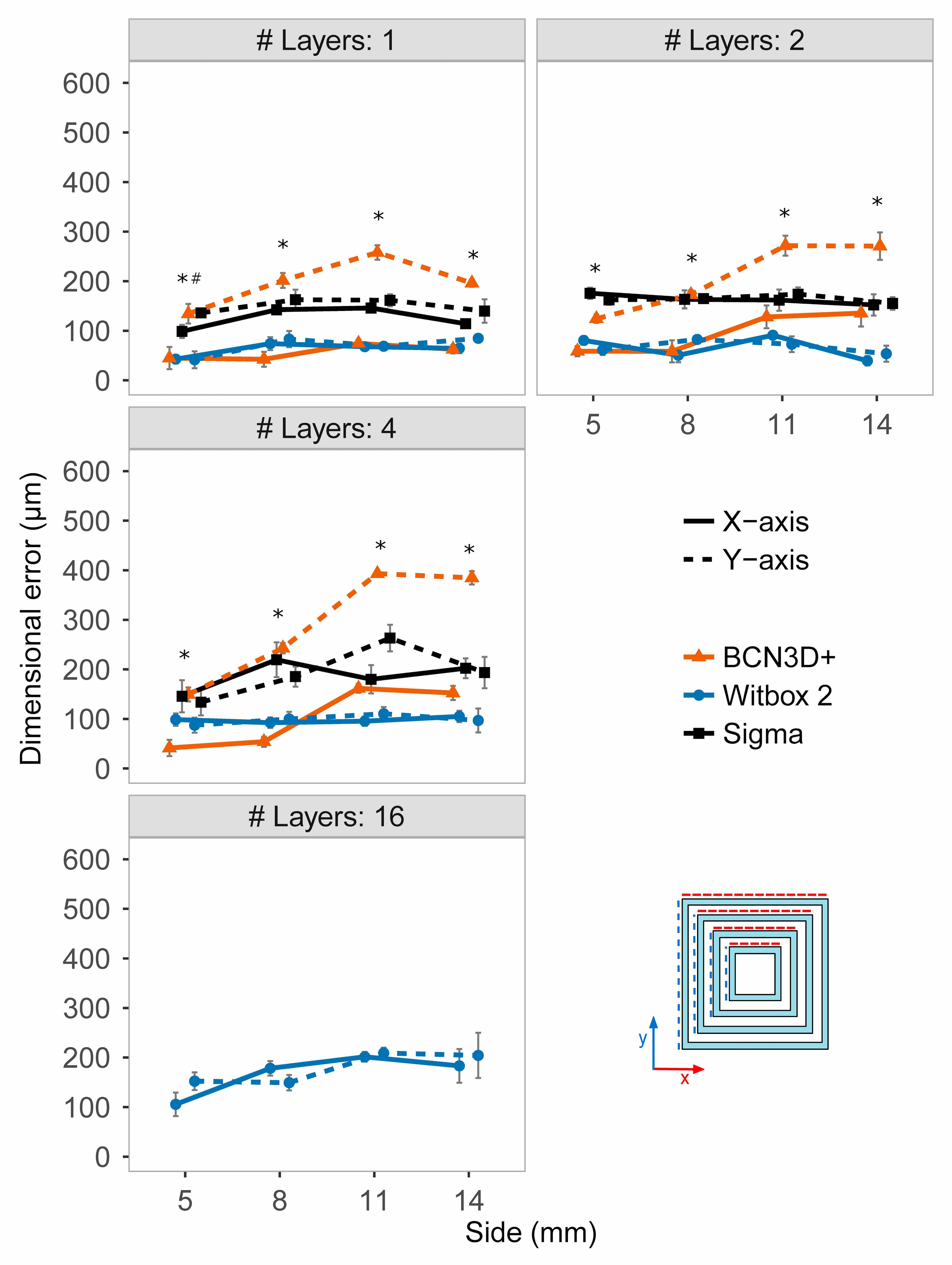

Supplement: Supplementary file 1 [file polymers-12-02346-s001.zip › polymers-972570-supplementary/Supporting Info/figS5.jpg]

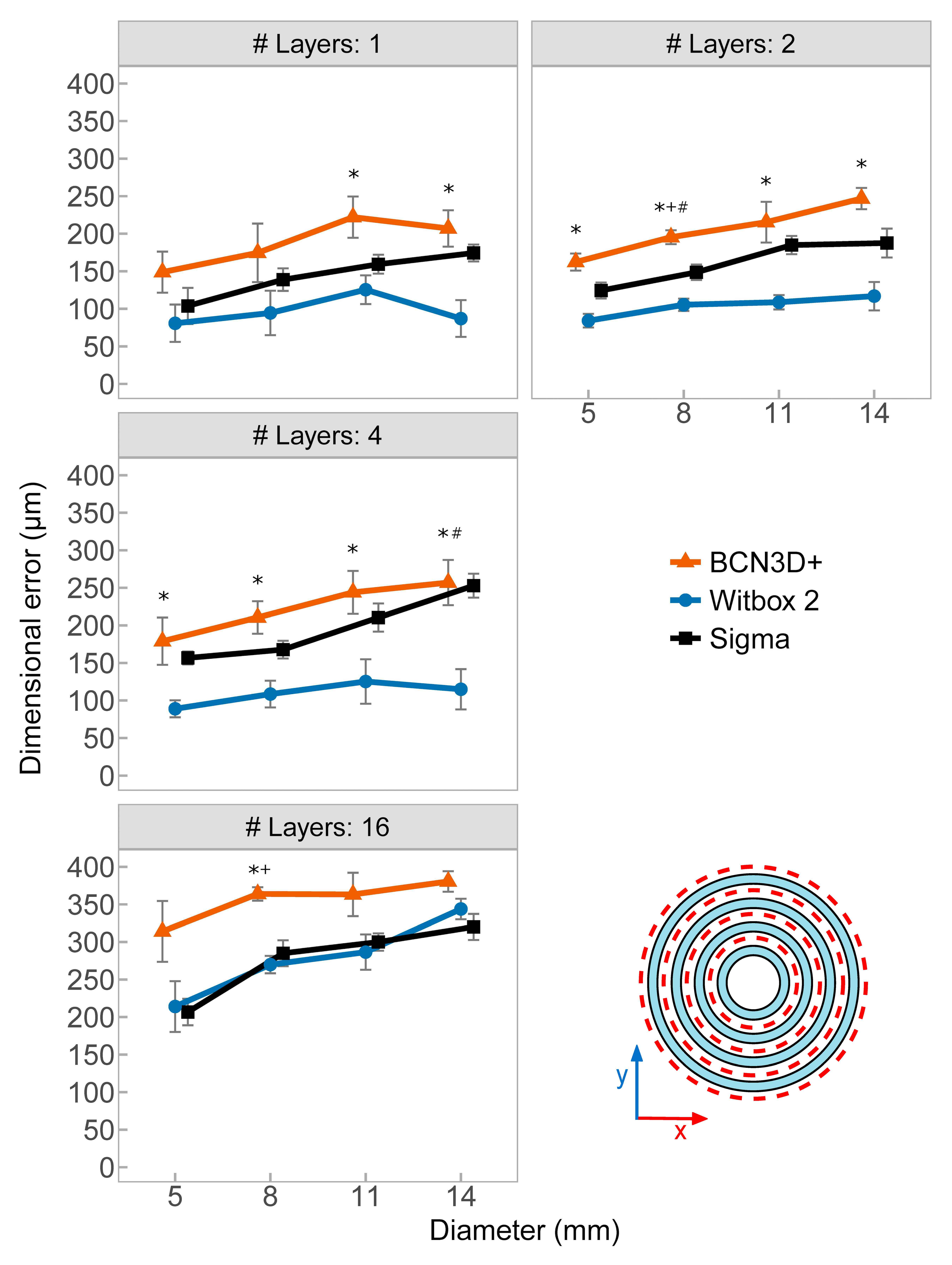

Supplement: Supplementary file 1 [file polymers-12-02346-s001.zip › polymers-972570-supplementary/Supporting Info/figS6.jpg]

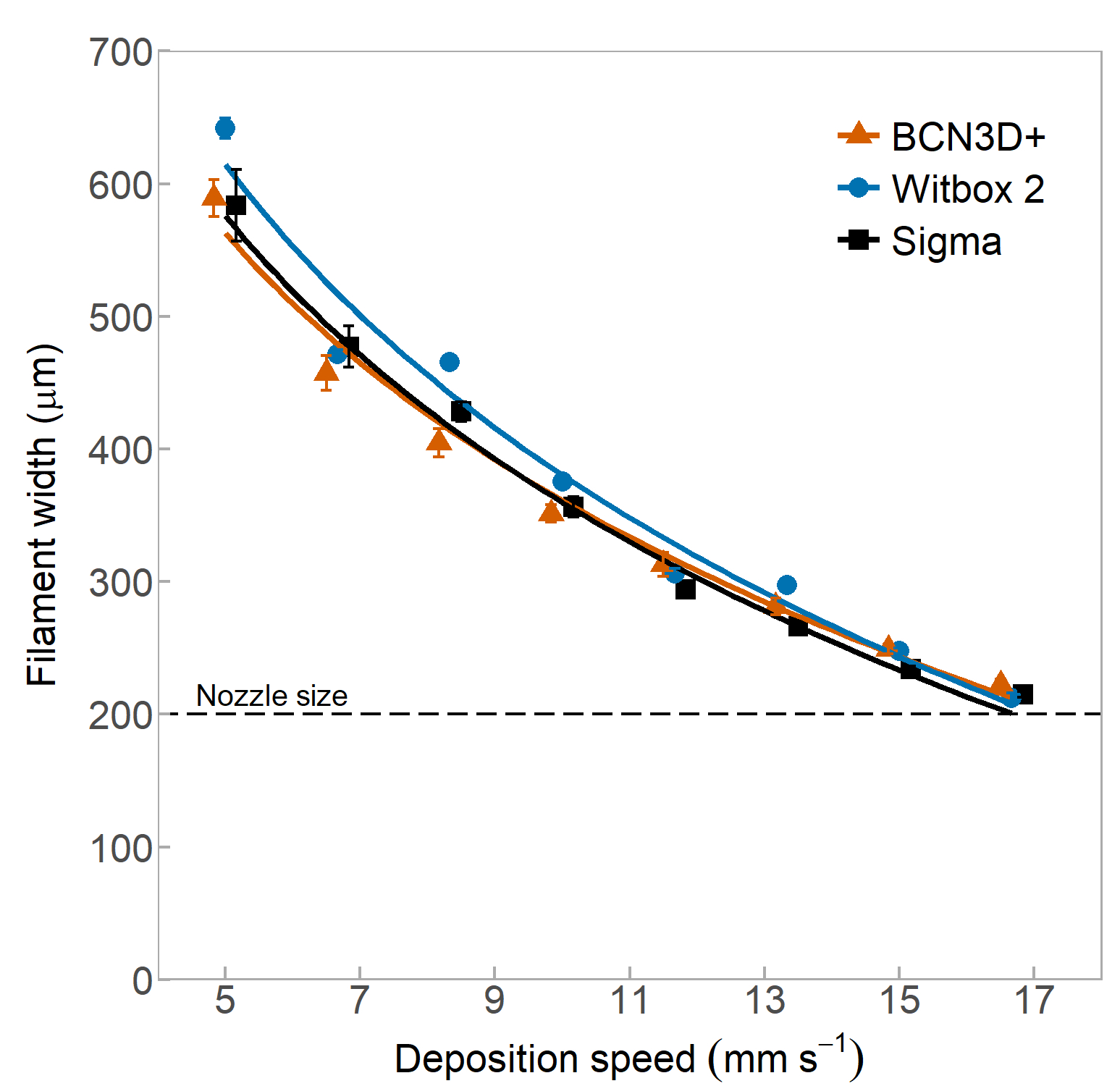

Supplement: Supplementary file 1 [file polymers-12-02346-s001.zip › polymers-972570-supplementary/Supporting Info/figS7.jpg]
